# Impact of Indoor Air Quality and Breathing on Back and Neck Pain: A Systematic Review

**DOI:** 10.7759/cureus.43945

**Published:** 2023-08-22

**Authors:** Ezequiel D Gherscovici, John M Mayer

**Affiliations:** 1 Research & Development, Healthy Buildings LLC, Malibu, USA

**Keywords:** spine, neck pain, back pain, exercises, musculoskeletal disorders, respiration, breathing, healthy buildings, indoor air quality, indoor environmental quality

## Abstract

Back pain and neck pain are important public health concerns and are among the most common and disabling conditions globally. However, the relationships among indoor air quality (IAQ), breathing parameters (pulmonary function, respiratory disorders), and back pain and neck pain have not been adequately assessed. The purpose of this study was to systematically review the literature about the impact of IAQ and breathing parameters on back pain and neck pain (PROSPERO ID: CRD42022380515). CINAHL, EMBASE, PEDRo, and PubMed databases were searched through January 19, 2023. Inclusion criteria for study eligibility were observational studies (except case reports) or randomized controlled trials (RCTs), published in peer-reviewed journals in the English language, human research, original research, examined the relationships between IAQ, or breathing parameters with back pain or neck pain. Review procedures were conducted and reported according to PRISMA recommendations. Empirical evidence statements were developed for observational studies, and grades of evidence statements were developed for RCTs. Sixty-seven eligible studies were found (54 observational studies and 13 RCTs) that enrolled 345,832 participants. None of the studies assessed the combined impact of IAQ and breathing parameters on back pain or neck pain. No level 1 studies were found, which precludes making strong statements about causality and strong recommendations about the efficacy of IAQ and breathing exercise interventions for reducing pain and disability related to back pain and neck pain. Evidence indicates that poor IAQ and respiratory disorders are related to an increased risk of back pain and neck pain. Conflicting evidence exists about the association between pulmonary function with back pain and neck pain. Evidence for breathing exercise interventions was mixed with numerous limitations. This review provides preliminary evidence on the relationships of IAQ and breathing parameters with back pain and neck pain, which can be used to guide future research and clinical implementation efforts. Assuming positive findings in subsequent research, a wide range of stakeholders involved with this complex human-building-environment interface can be equipped to address IAQ and breathing parameters, along with other established risk factors to help those suffering from back pain and neck pain.

## Introduction and background

Back pain and neck pain are important public health concerns and are among the most common, costly, and disabling conditions in the world [1-6]. In the global burden of disease (GBD) studies [2,6,7], low back pain (LBP) is the most common cause of years lived with disability (YLDs) and a leading cause of disability-adjusted life years (DALYs), and neck pain is also problematic in terms of YLDs and DALYs. Most adults will experience disabling LBP or neck pain at some point in their lives [3,4], and symptoms and disability often persist for those who suffer initial episodes [4,5].

Numerous biopsychosocial risk factors have been identified for back pain and neck pain, such as age, previous history of the condition, obesity, sub-optimal fitness, low physical activity, psychological conditions, smoking, poor ergonomics, and awkward lifting [4,5]. Our recent systematic reviews found additional risk factors for back pain, neck pain, and other musculoskeletal disorders (MSDs) within the built environment that were not previously identified and classified as healthy building determinants (HBDs) [8,9]. For example, evidence was found to support an association between sub-optimal indoor air quality (IAQ) and increased risk of back pain and neck pain. However, the available evidence was primarily from lower-level studies; thus, conclusions about causality and intervention effectiveness could not be made. Others have found that various breathing parameters (e.g., pulmonary function, respiratory disorders) are associated with LBP and neck pain [10,11]. Yet, the inter-relationships of IAQ and breathing parameters on back pain and neck pain have not been adequately explored.

Among the various treatment options for back pain and neck pain, the clinical practice guidelines (CPGs) generally recommend therapeutic exercises to improve pain, disability, and function for managing these disorders [1,3,12]. While many different types of exercises are available for the management of back pain and neck pain, no specific type has been shown to be clearly superior to others [13,14]. With some exceptions, such as directional preference exercises through mechanical diagnosis and therapy [15,16], exercises have generally been studied for heterogeneous groups of patients with LBP and neck pain [17]. Thus, the available evidence is unclear about how to match the right patient with the right intervention at the right time.

Given the relationships of IAQ and breathing parameters with back pain and neck pain, along with the lack of clarity on which exercises are best for specific patients, it is plausible that interventions aimed at improving IAQ and breathing parameters, along with the array of other established risk factors, may be useful for reducing the risk of these disorders in public, residential, and workplace environments. While our previous reviews did not find any RCTs examining the efficacy of IAQ interventions on various MSDs [8,9], a recent systematic review found preliminary evidence from small randomized controlled trials (RCTs) to support the efficacy of breathing exercises on short-term pain measures for LBP [18]. Another systematic review on this topic uncovered one small RCT that assessed the independent effect of breathing exercises compared to non-breathing exercise control for LBP [19]. Additionally, a systematic review of exercise for neck pain found one small RCT on breathing exercises that did not support efficacy of this intervention [13].

Conceivably, addressing both IAQ and breathing parameters at the same time within the human-building-environment interface may be useful in reducing the risk of back pain and neck pain. For example, previous research indicates that poor IAQ contributes to tissue hypoxia [20] and is related to sick building syndrome [21], which is associated with MSDs [21]. Improving IAQ by addressing a building's air filtration and ventilation systems can decrease exposure to six common exterior air pollutants (i.e., ground-level ozone, particulate matter, carbon monoxide, lead, sulfur dioxide, nitrogen dioxide) that are known to affect human health [22]. Furthermore, disordered breathing is associated with abnormal carbon dioxide and oxygen physiology [23] and reduced functional movement quality [24], which is related to increased risk for MSDs [23,25]. Improving breathing patterns, such as the use of nose breathing and the light, slow, and deep technique [26,27], can enhance the body's ability to filter air and recover [26,27]. However, research on the combined effect of IAQ and breathing parameters on back pain and neck pain has not been systematically examined. The purpose of this study was to systematically review the literature on the impact of IAQ and breathing parameters on back pain and neck pain.

## Review

Materials and methods

Overview

The current review incorporated similar methods, evidence synthesis procedures, and reporting structure as our earlier reviews that examined HBDs and MSDs [8,9]. The current review was conducted and reported according to the Preferred Reporting Items for Systematic Reviews and Meta-Analyses (PRISMA) [28] and other resources [1,12,29-34] and was registered with PROSPERO (ID: CRD42022380515).

Information Sources

Studies were uncovered by searching CINAHL, EMBASE, PEDRo, and PubMed. The last author (JM) developed the search strategy, and the first author (EG) cross-checked it. The PubMed search strategy is shown in the Appendices, and CINAHL, EMBASE, and PEDRo were searched using a comparable database-specific approach. To identify additional studies, hand searches of published reports available to the authors were conducted, and an examination of references within studies obtained from the primary search was performed [8,9].

Eligibility Criteria

Inclusion and exclusion criteria are depicted using the PICOTS method: P - patients/people, I - intervention, C - comparator, O - outcomes/variables, T - time/timing, S - setting [8,9,28].

P - Patients/people: Studies were included if they assessed humans ≥ 18 years of age with back pain, neck pain, or related MSDs (e.g., cervical radiculopathy, lumbar radiculopathy, sciatica). Back pain is defined as pain or associated symptoms in the thoracic spine region [35] or the lumbo-sacral spine region [36]. Neck pain is defined as pain or associated symptoms in the cervical spine region [35,37]. Studies were included that described all forms, severities, and durations of back pain and neck pain. Studies were excluded that only described systemic disorders (e.g., fibromyalgia) or neurological conditions (e.g., multiple sclerosis) [8,9].

I - Intervention: Studies were included if they examined IAQ or breathing parameters (pulmonary function, respiratory disorders). For the purpose of this review, IAQ consisted of air quality and ventilation HBDs, which are defined elsewhere [38]. Definitions for constructs related to IAQ are found elsewhere for healthy buildings [8,39], built environments [40], determinants of health [41], and HBDs [8]. For the purpose of this review, the breathing parameters of pulmonary function and respiratory disorders were included. Pulmonary function (i.e., lung function) is defined as "...how well the lungs work in helping a person breathe. During breathing, oxygen is taken into the lungs, where it passes into the blood and travels to the body’s tissues. Carbon dioxide, a waste product made by the body’s tissues, is carried to the lungs, where it is breathed out. There are different tests to measure pulmonary function" [42]. Respiratory disorders (i.e., respiratory diseases) are defined as "…any of the diseases and disorders of the airways and the lungs that affect human respiration," such as asthma and chronic obstructive pulmonary disease (COPD) [43]. Breathing exercises are defined as a type of exercise specifically designed to enhance the respiratory system by improving ventilation, strengthening respiratory muscles, and making respiration more efficient [44]. Breathing exercises should focus on the therapeutic goal of proper breathing mechanics for the required metabolic demands and desired outcomes [26]. Several factors should be considered for breathing exercises, including various combinations of nose and mouth breathing during inhalation and exhalation, frequency, speed, cadence, and depth of breathing. For most people, "proper" breathing during the resting state should be inhalation and exhalation through the nose in a controlled, light, slow, and deep manner [26,27]. RCTs were included that prospectively examined the efficacy of IAQ interventions or breathing exercise interventions on back pain or neck pain. For the RCTs, the independent effect of an IAQ intervention or breathing exercise intervention on back pain or neck pain must have been assessed, regardless of whether that intervention was delivered alone or in combination with other interventions. Studies were excluded in which breathing may have been a component of multifaceted interventions, such as cardiovascular exercises, Yoga, Tai Chi, Qigong, and Pilates.

C - Comparator: Studies were eligible for inclusion if they compared the previously described IAQ or breathing parameters with back pain or neck pain. As previously mentioned, the independent impact of IAQ or breathing on back pain or neck pain must have been assessed, regardless of whether that intervention was delivered alone or combined with other interventions. For the case-control studies and RCTs, control groups must have been distinct from the cases or active intervention and adequately described.

O - Outcomes/variables: Studies were included if they used various strategies to assess IAQ, breathing parameters (pulmonary function, respiratory disorders), and back pain and neck pain, such as patient-reported, physical, functional, and environmental outcome measures. Studies were included that examined measures directly associated with back pain and neck pain, for example, pain, disability, and lost work time. Studies were excluded that only examined outcomes indirectly related to back pain and neck pain, such as body mass index, muscular characteristics, and psychosocial measures [8,9]. For the RCTs on breathing exercises, studies were included if they assessed pain or disability outcomes [16,45]. If the RCT assessed pain or disability, then pulmonary function outcomes were also considered.

T - Time/timing: Studies were included if they were published in peer-reviewed journals and indexed from database inception through January 19, 2023.

S - Setting: Studies were included if they assessed an IAQ parameter or an IAQ intervention within the indoor built environment of commercial, public, residential, or work-related real estate settings. Studies were excluded that assessed the air quality of outdoor settings. Studies were included that assessed a breathing parameter in any setting. For breathing exercise interventions, RCTs were included if they reported on interventions for back pain or neck pain that were delivered in any setting. For back pain or neck pain, studies were included if they reported on these conditions, or their management, in any setting.

Additionally, studies were included if they were published in a peer-reviewed journal and in the English language, human research, the abstract was available for preliminary screening, and the full-text article was available for the final determination processes. Except for case reports, all types of subject-level original research studies were included, such as observational studies (e.g., cohort, case-control, cross-sectional) and controlled trials. Studies were excluded that were non-human studies (e.g., animal, basic science, laboratory, or simulation), grey literature, and reviews [8,9].

Data Extraction

Study selection: Search results were handled using a citation manager and spreadsheet databases [8,9]. After preliminary management of the extracted articles, EG and JM separately screened citations (e.g., title, abstract) to assess eligibility. Articles were initially classified as relevant, possibly relevant, or irrelevant. Subsequent to reaching a final consensus, full-text PDFs were acquired for articles considered to be relevant or possibly relevant. EG and JM separately screened the full-text articles for relevance. Then, the two authors worked together to reach a final consensus on the eligible articles. Automation processes were not utilized to select articles [8,9].

Data extraction: JM extracted data from the eligible articles and entered them into a database, and EG separately cross-checked the results [8,9]. Then, they worked together until reaching a consensus regarding the extracted data. Automation was not utilized for data extraction. Data that were entered into the tables included author, year, country, funding source, population, sample size, gender, age, eligibility criteria, factors examined (IAQ, pulmonary function, respiratory disorder, back pain, neck pain), outcomes measured, case and control details (for case-control studies), intervention and control details (for RCTs), analysis procedures, and results. Missing data were not considered in the evidence synthesis procedures and are reported in the tables accordingly. If necessary, the authors of the original articles were contacted by email to clarify the study findings [8,9].

Data Synthesis

Overview: Approaches adapted from the Oxford Centre for Evidence-Based Medicine, Clinical Information Access Portal [29-32], and American Physical Therapy Association [1,12] were used to handle data and synthesize evidence [8,9].

Study quality (risk of bias): Study quality was assessed using the NIH quality assessment instruments for observational studies and controlled trials [46]. As shown in the Appendices, these instruments have 14 items, in which each item is rated as Yes = 1 or No = 0, to derive a total instrument score from 0 to 14 [46]. Ranges from the total score were used to derive study quality categories as follows: 0-4 = Poor quality (high risk of bias), 5-9 = Fair quality (between low risk and high risk of bias), and 10-14 = Good quality (low risk of bias) [8,9,46].

Level of evidence (study type): The level of evidence was determined using approaches adapted from the Oxford Centre for Evidence-Based Medicine [29-32]. JM assessed the study quality and evidence level, and EG separately cross-checked the results. Subsequently, the two authors worked together until reaching a consensus about study quality and evidence level. Automation was not utilized to assess study quality and evidence level. Reporting bias was not assessed, and missing data are reported in the tables [8,9].

Evidence synthesis: Empirical evidence statements (for relationships assessed in observational studies) and the grade of evidence statements (for interventions assessed in RCTs) were synthesized based on strategies adapted from the Oxford Centre for Evidence-Based Medicine [30-32], American Physical Therapy Association [1,12], and relevant systematic reviews [8,9,34].

Empirical evidence statements from observational studies were based on pairwise comparisons of IAQ, pulmonary function, or respiratory disorders with back pain or neck pain (six total pairwise comparisons). The grade of evidence statements from RCTs was based on pairwise comparisons of breathing exercise interventions (alone or combined with other interventions) for back pain or neck pain across pain, disability, and pulmonary function outcomes (12 total pairwise comparisons). Empirical evidence statements and grade of evidence statements for the pairwise comparisons were developed utilizing the categories shown below, which were adapted from other work [1,12] and our previous reviews [8,9]. Adaptations made to the quoted statements for the purposes of the current review are shown in brackets. (1) Strong evidence: "One or more level I systematic reviews (or studies) support the recommendation" [1]. (2) Moderate evidence: "One or more level II systematic reviews (or studies) or a preponderance of level III systematic reviews or studies support the recommendation" [1]. (3) Weak evidence: "One or more level III systematic reviews (or studies) or a preponderance of level IV evidence supports the recommendation [1]. (4) Conflicting evidence: "… studies conducted on this topic disagree with respect to their conclusions and effect" [1]. (5) Insufficient evidence: "Too few studies of fair to good quality exist to draw conclusions" [47], or evidence is available from only one lesser quality RCT, which is defined by improper randomization, or no blinding, or less than 80% follow-up [12]. (6.) No evidence is available.

Meta-analysis, heterogeneity analysis, and sensitivity analysis were not carried out for the following reasons:

1. The available evidence and outcome measured assessed varied widely, did not include any level I evidence, or was limited for certain comparisons and, therefore, was not conducive to such analyses.

2. The studies enrolled people with heterogeneous types of back pain and neck pain without considering precise diagnostic or treatment classifications.

3. Among the RCTs, the breathing exercise interventions administered were heterogenous and standardized approaches were not used.

Results

Study Selection

**Figure 1:**
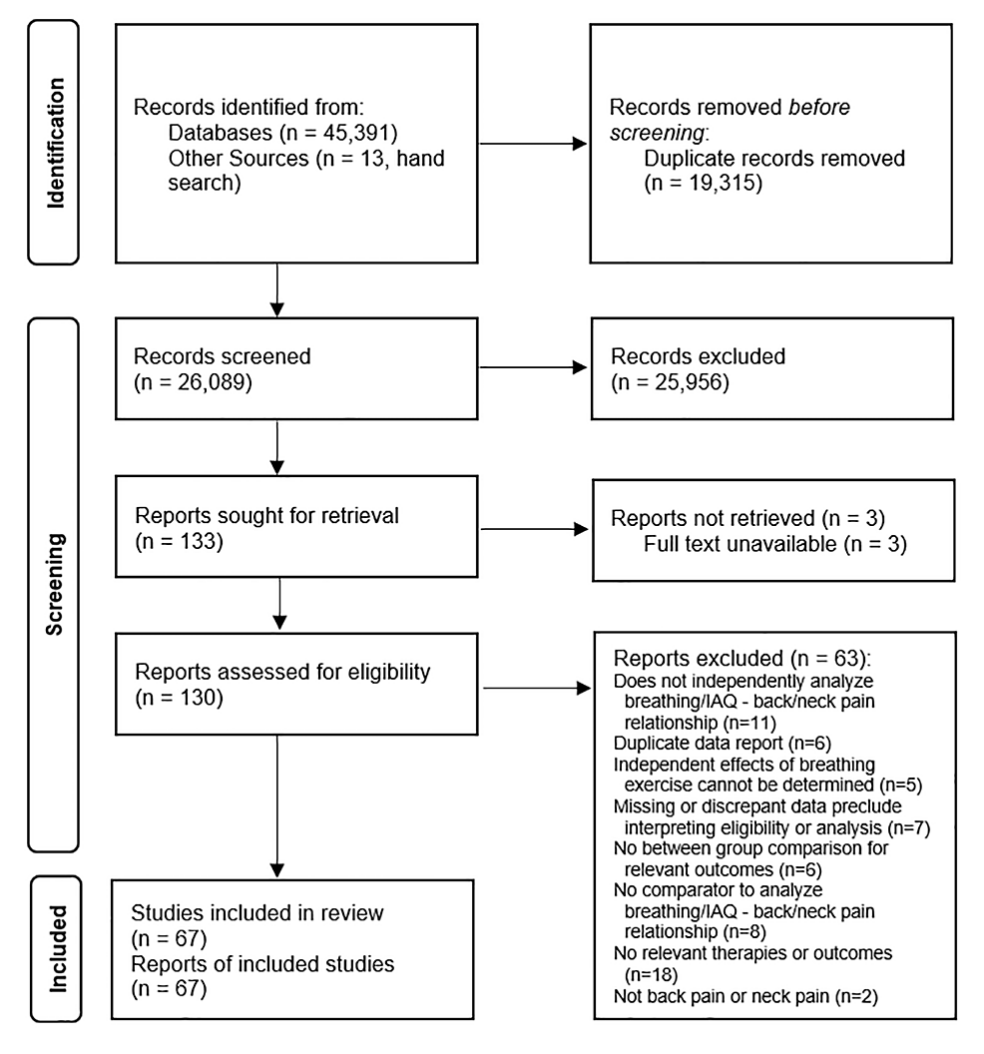
PRISMA flow diagram of search results

Search results are found in the PRISMA diagram (Figure 1). Sixty-seven eligible studies that enrolled 345,832 participants were uncovered [48-114].

Sixty-three studies appeared to be eligible at preliminary review, but were deemed ineligible for the following reasons: Does not independently analyze breathing/IAQ - back/neck pain relationship (N = 11) [115-125], duplicate data report (N = 6) [126-131], independent effects of breathing exercise that cannot be determined (N = 5) [132-136], missing or discrepant data that preclude interpreting eligibility or analysis (N = 7) [137-143], no between-group comparison for relevant outcomes (N = 6) [144-149], no comparator to analyze breathing/IAQ - back/neck pain relationship (N = 8) [150-157], no relevant therapies or outcomes (N = 18) [158-175], and not back pain or neck pain (N = 2) [176,177].

Study Characteristics

Details of the characteristics and outcomes of the included studies are shown in the Appendices. None of the included studies assessed the combined impact of IAQ and breathing parameters on back pain or neck pain. Fifty-two studies assessed the general population in unspecified settings [48-50,52,53,55,58-64,67,68,70-77,79-85,87,89,90,92-94,96-102,104-109,111-113]. Thirteen studies assessed workers in various occupational settings [51,54,56,57,65,69,78,86,88,95,103,110,114]. Two studies assessed the general population in residential settings [66,91]. The studies were conducted in 28 countries as follows: Australia (N = 6) [53,63,84,93,106,107], Belgium (N = 2) [56,104], Brazil (N = 1) [87], Canada (N = 2) [57,83], China (N = 3) [59,78,114], Denmark (N = 2) [76,100], Egypt (N =1) [52], Finland (N = 1) [110], France (N = 1) [75], Greece (N = 3) [67,68,79], Guatemala (N = 1) [66], India (N = 4) [80,102,103,105], Iran (N = 6) [48,55,62,72,91,94], Iraq (N = 1) [70], Italy (N = 1) [88], Korea (N = 7) [58,92,95-99], Malaysia (N = 1) [90], Norway (N = 1) [69], Pakistan (N = 1) [50], the Philippines (N = 1) [86], Spain (N = 3) [64,71,85], Sweden (N = 4) [51,74,101,108], Switzerland (N = 2) [49,111], Thailand (N = 1) [54], Turkey (N = 4) [61,81,109,113], United Kingdom (N = 2) [65,112], and United States (N = 5) [60,73,77,82,89]. The publication years of the studies ranged from 1981 through 2023, with 58% (39/67) published in the past 10 years (since 2014), as follows: 1980-1989 (N = 4) [51,57,65,108], 1990-1999 (N = 4) [77,78,110,112], 2000-2009 (N = 13) [56,59,66,69,75,76,79,86,89,93,104,106,107], 2010-2019 (N = 25) [49,60,63,64,67,68,73,74,83-85,87,88,90,91,95,96,100-103,105,111,113,114], and 2020-2023 (N = 21) [48,50,52-55,58,61,62,70-72,80-82,92,94,97-99,109]. The funding sources for the studies were as follows: extramural (e.g., academic, government, non-profit, commercial) (N = 26) [51,53,54,59,60,63,65,69,71,73,76,84,89,91,94-98,100,103,104,106-108,113], internal (no extramural funding) (N = 11) [50,52,61,62,70,72,80,99,102,105,109], and not reported (N = 30) [48,49,55-58,64,66-68,74,75,77-79,81-83,85-88,90,92,93,101,110-112,114].

For the 54 observational studies, back pain alone was assessed in 30 studies [51,53,59,60,65,73,75-77,81,82,84,86,88,90,93,97,99-102,104-110,112,114]. Neck pain alone was assessed in 12 studies [52,54,56,58,61,67,68,70,79,85,111,113]. Both back pain and neck pain were assessed in 12 studies [49,57,63,64,69,71,78,83,87,91,95,103]. IAQ was assessed in 13 studies [56,57,69,78,86,88,91,95,100,103,109,110,114]. Pulmonary function was assessed in 19 studies [52,54,58,61,65,67,68,70,73,75,79,85,90,93,102,104,105,111,113]. Respiratory disorders were assessed in 22 studies [49,51,53,59,60,63,64,71,76,77,81-84,87,97,99,101,106-108,112].

For the 13 RCTs, back pain alone was assessed in 10 RCTs [48,55,66,72,80,89,92,94,96,98]. Neck pain alone was assessed in three RCTs [50,62,74]. One RCT assessed the impact of an IAQ intervention on long-term back pain or neck pain outcomes [66]. Twelve RCTs assessed the impact of breathing exercise interventions on back pain or neck pain outcomes [48,50,55,62,72,74,80,89,92,94,96,98]. The sample size enrolled in the 12 RCTs assessing breathing exercises was relatively small, enrolling a mean (SD, range) of 43.9 (13.0, 24-68) participants. One of the RCTs assessed longer-term (i.e., six-month) outcomes [89], while the remaining 11 RCTs assessed short-term outcomes. Chronic (≥ 3 months duration) back or neck pain was assessed in nine RCTs [48,50,55,62,74,80,89,92,94]. Sub-acute/chronic (≥ six weeks duration) back or neck pain was assessed in two RCTs [96,98], while condition duration was not reported in one RCT [72]. The type of breathing exercises varied widely across the RCTs; thus, it was not possible to group the breathing exercises into standardized categories. Breathing exercises were administered in combination with other interventions in nine RCTs [50,62,72,80,89,92,94,96,98] and were administered alone in three RCTs [48,55,74]. Physical therapists delivered breathing exercises in 10 RCTs [48,50,55,62,72,80,92,94,96,98]. A psychologist delivered breathing exercises in one RCT [74], and a breath therapist delivered breathing exercises in one RCT [89].

Study Outcomes

For the included observational studies, the outcome measures were primarily descriptive and relational [8,9] and varied widely across study types, as well as the IAQ, breathing, and respiratory disorders. Outcomes for IAQ were mainly study-specific and not validated for general use. Outcomes for breathing were wide-ranging, many of which were validated pulmonary function tests (e.g., forced vital capacity). Outcomes for respiratory disorders were primarily prevalence and incidence. The observational studies also used various outcome measures for back and neck pain, such as validated patient-reported outcomes (e.g., Nordic musculoskeletal questionnaire) and administrative measures (e.g., work absenteeism), as well as study-specific measures that have not been validated. For the RCTs, validated outcome measures for pain (e.g., visual analog scale), disability (e.g., Oswestry disability index), and pulmonary function tests (e.g., forced vital capacity) were used.

Evidence Level and Study Quality

Study level and quality are shown in the Appendices. The evidence level of the eligible studies was as follows: Level 2 (RCTs) (N = 13) [48,50,55,62,66,72,74,80,89,92,94,96,98], Level 2 (prospective observational cohort) (N = 9) [49,53,60,65,69,76,101,107,110], Level 3 (case-control) (N = 24) [52,54,58,61,64,67,68,70,71,73,75,79,83,85,87,90,93,95,102-105,111,113], Level 3 (retrospective cohort) (N = 1) [97], and Level 4 (cross-sectional) (N = 20) [51,56,57,59,63,77,78,81,82,84,86,88,91,99,100,106,108,109,112,114]. The mean (SD, range) study quality score for the eligible studies was 7.5 (2.0, 2-11). Study quality categories were as follows: Good (N = 14) [48,53,55,62,65,69,72,76,80,92,97,98,107,110], Fair (N = 50) [49-52,54,56,58-61,63,64,66-68,70,71,73-75,77,79,81-85,87-91,93-96,99-106,108,109,111-114], and Poor (N = 3) [57,78,86]. No level 1 studies were found (e.g., higher quality RCTs), which precludes making strong empirical evidence statements, grade of evidence statements, confirmatory interpretations about causal relationships, and conclusions about the efficacy of IAQ and breathing exercise interventions for reducing pain and disability related to back pain and neck pain.

Empirical Evidence Statements - Observational Studies

Empirical evidence statements from observational studies for the relationships of IAQ, pulmonary function, and respiratory disorders with back pain and neck pain are detailed in Table 1. This review found evidence to support significant weak relationships between IAQ and back pain, based on 12 studies for back pain and seven studies for neck pain. Namely, poor IAQ is related to an increased risk of back pain and neck pain. Similarly, this review found evidence to support significant moderate relationships between various respiratory disorders and back pain and neck pain, based on 22 studies for back pain and six studies for neck pain. That is, the presence of respiratory disorders is associated with an increased risk of back pain and neck pain. On the contrary, this review found conflicting evidence about the relationships between pulmonary function and back pain and neck pain, based on eight studies for back pain and 11 studies for neck pain. Many studies examining these relationships reported mixed results among various pulmonary function measures. That is, some results support a relationship between pulmonary function and back pain and neck pain, while some results do not support a relationship.

**Table 1:** Empirical evidence statements from observational studies for the relationship of indoor air quality, pulmonary function, and respiratory disorders with back pain and neck pain. Yes: Results of all study outcome measures support the relationship between outcome and back pain or neck pain. Mixed: Results of study outcome measures are mixed: some results support the relationship between outcome and back pain or neck pain, and some results do not support the relationship. No: Results of all study outcome measures do not support the relationship between outcome and back pain or neck pain.

Outcome	Back Pain	Neck Pain
Indoor Air Quality	Weak evidence from 12 studies indicates that poor indoor air quality is associated with an increased risk of back pain. Yes: [69,88,91,100,103]. Mixed: [57,95,109,110]. No: [78,86,114].	Weak evidence from 7 studies indicates that poor indoor air quality is associated with an increased risk of neck pain. Yes: [56,69,91,95]. Mixed: [57,103]. No: [78].
Pulmonary Function	Conflicting evidence from 8 studies exists about the association between pulmonary function and back pain. Yes: [102]. Mixed: [65,73,93,104,105]. No: [75,90].	Conflicting evidence from 11 studies exists about the association between pulmonary function and neck pain. Yes: [52,54]. Mixed: [58,61,67,68,70,79,85]. No: [111,113].
Respiratory Disorders	Moderate evidence from 22 studies indicates that the presence of respiratory disorders is associated with an increased risk of back pain. Yes: [49,53,59,60,63,64,71,76,77,81,87,97,99,101,106-108,112]. Mixed: [51,82,83]. No: [84].	Moderate evidence from 6 studies indicates that the presence of respiratory disorders is associated with an increased risk of neck pain. Yes: [49,63,64,71,87]. Mixed: none. No: [83].

Grade of Evidence Statements - RCTs

The grade of evidence statements from RCTs assessing the efficacy of breathing exercise interventions on back pain and neck pain is detailed in Table 2. For back pain, moderate evidence from two RCTs indicates that breathing exercises alone, compared to control, may be useful to improve pain outcomes. Conflicting evidence from seven RCTs exists about the efficacy of adding breathing exercises to another intervention compared to that intervention alone on pain outcomes. Insufficient evidence from one RCT is available to assess the efficacy of breathing exercises alone compared to control on disability outcomes. Conflicting evidence from seven RCTs exists about the efficacy of adding breathing exercises to another intervention compared to that intervention alone on disability outcomes. Insufficient evidence from one RCT is available to assess the efficacy of breathing exercises alone compared to control on pulmonary function outcomes. Conflicting evidence from four RCTs exists about the efficacy of adding breathing exercises to another intervention compared to that intervention alone on pulmonary outcomes.

**Table 2:** Grade of evidence statements from randomized controlled trials for the efficacy of breathing exercises on short-term pain, disability, and pulmonary function outcomes for the management of back pain and neck pain. Yes: Results of all study outcome measures support breathing exercises. Mixed: Results of study outcome measures are mixed: some results support breathing exercises, and some results do not support breathing exercises. No: Results of all study outcome measures do not support breathing exercises. RCT: Randomized Controlled Trial.

Outcome	Back Pain	Neck Pain
Pain	Moderate evidence from two RCTs indicates that breathing exercises alone, compared to control, may be useful to improve pain outcomes. Yes: [48,55]. Mixed: none. No: none. Conflicting evidence from seven RCTs exists about the efficacy of adding breathing exercises to another intervention compared to that intervention alone on pain outcomes. Yes: [72,94,98]. Mixed: [80]. No: [89,92,96].	Insufficient evidence from one RCT is available to assess the efficacy of breathing exercises alone compared to control on pain outcomes. Yes: none. Mixed: none. No: [74]. Conflicting evidence from two RCTs exists about the efficacy of adding breathing exercises to another intervention compared to that intervention alone on pain outcomes. Yes: [50]. Mixed: none. No: [62].
Disability	Insufficient evidence from one RCT is available to assess the efficacy of breathing exercises alone compared to control on disability outcomes. Yes: none. Mixed: none. No: [55]. Conflicting evidence from seven RCTs exists about the efficacy of adding breathing exercises to another intervention compared to that intervention alone on disability outcomes. Yes: [72,92,98]. Mixed: none. No: [80,89,94,96].	Insufficient evidence from one RCT is available to assess the efficacy of breathing exercises alone compared to control on disability outcomes. Yes: none. Mixed: none. No: [74]. Insufficient evidence from one RCT is available to assess the efficacy of adding breathing exercises to another intervention compared to that intervention alone on disability outcomes. Yes: [50]. Mixed: none. No: none.
Pulmonary Function	Insufficient evidence from one RCT is available to assess the efficacy of breathing exercises alone compared to control on pulmonary function outcomes. Yes: [48]. Mixed: none. No: none. Conflicting evidence from four RCTs exists about the efficacy of adding breathing exercises to another intervention compared to that intervention alone on pulmonary outcomes. Yes: [72]. Mixed: [92,96,98]. No: none.	No evidence is available to assess the efficacy of breathing exercises alone compared to control on pulmonary function outcomes. Moderate evidence from two RCTs indicates that adding breathing exercises to another intervention compared to that intervention alone may be useful to improve pulmonary function outcomes. Yes: [50,62]. Mixed: none. No: none.

The available evidence on breathing exercise interventions for neck pain was likewise mixed. Insufficient evidence from one RCT is available to assess the efficacy of breathing exercises alone compared to control on pain outcomes. Conflicting evidence from two RCTs exists about the efficacy of adding breathing exercises to another intervention compared to that intervention alone on pain outcomes. Insufficient evidence from one RCT is available to assess the efficacy of breathing exercises alone compared to control on disability outcomes. Insufficient evidence from one RCT is available to assess the efficacy of adding breathing exercises to another intervention compared to that intervention alone on disability outcomes. No evidence is available to assess the efficacy of breathing exercises alone compared to control on pulmonary function outcomes. Moderate evidence from two RCTs indicates that adding breathing exercises to another intervention compared to that intervention alone may be useful to improve pulmonary function outcomes.

For IAQ, insufficient evidence from one RCT is available to assess the efficacy of an IAQ intervention compared to control on pain and pulmonary function outcomes for back pain. No evidence is available to assess the efficacy of IAQ interventions on disability outcomes for back pain. Moreover, no evidence is available to assess the efficacy of IAQ interventions on pain, disability, and pulmonary function outcomes for neck pain.

Discussion

The findings of the current review add to the body of knowledge on the impact of IAQ and breathing parameters on back pain and neck pain. When the current review is considered along with other recent efforts, the available evidence provides a comprehensive preliminary assessment of this topic that can be used to inform future research and implementation initiatives. Overall, this review found 67 studies (54 observational studies and 13 RCTs) on relationships of IAQ or breathing parameters with back pain or neck pain. More than half (39/67) of these studies were published over the past decade (since 2014) and were conducted in a wide range of countries, settings, and populations, which suggests that the interest in this topic is growing. The uncovered studies provide preliminary evidence on the relationships of IAQ or breathing parameters with back pain and neck pain, which can be used to guide future research and clinical implementation efforts. Key findings are as follows:

1. None of the uncovered studies assessed the combined impact of IAQ and breathing parameters on back pain or neck pain.

2. Evidence indicates that IAQ and respiratory disorders are associated with back pain and neck pain, which is consistent with previous work.

3. Conflicting evidence exists about the association between pulmonary function with back pain and neck pain.

4. Evidence for breathing exercise interventions is mixed with numerous limitations, which precludes making strong recommendations for or against their use for reducing pain and disability related to back pain and neck pain.

Contrary to our assumptions before conducting this review, no studies were found that examined the combined impact of IAQ and breathing parameters on back pain and neck pain. Furthermore, our anecdotal observations outside of this review suggest that no clinical programs or commercial initiatives have implemented strategies to address both factors in people with back pain and neck pain. We speculate that a primary reason to explain no research or program implementation efforts is the lack of awareness and disconnect among the diverse stakeholders involved with decision-making about these relationships. As we previously described [8], the stakeholder sectors include (1) healthcare (e.g., patients, clinicians, managed care organizations), (2) real estate (e.g., tenants, owners, investors, property managers, engineers, architects), (3) occupational (e.g., employees, employers), (4) policy (e.g., regulatory, licensing, credentialing), and (5) public health (e.g., public health officials and organizations). Considering the large magnitude of tackling the combined impact of IAQ and breathing parameters on back pain and neck pain, it is possible that each stakeholder is working in silos and approaching the problem with their unique point of view. In our opinion, a better approach would be working together to address known risk factors with a common goal of reducing the adverse effects of back pain and neck pain. We acknowledge that reaching a consensus among so many interested parties is challenging because it is impossible for a specific stakeholder group to be well-versed in the field at large. Moreover, stakeholder-specific biases and conflicts of interest add other barriers. Some of these explanations have been mentioned as problematic for the overall management of LBP [178]. Regardless, the findings of the current review can serve to enhance awareness and provide a framework to help guide future efforts.

For the observational studies, the findings about IAQ in the current review confirm those from our previous review [8], which indicates that poor IAQ is associated with an increased risk of back pain and neck pain. While more studies were found in the current review (13 in the current review vs. 10 in a previous review), the uncovered studies were primarily lower level, and the updated findings continue to support a weak association. The findings on the association of respiratory disorders with back pain are mostly consistent with another review on this topic [10]. Namely, the presence of respiratory disorders is associated with an increased risk of back pain. We did not find another review to compare findings about the association of respiratory disorders with neck pain. The current review found conflicting evidence about the associations between pulmonary function and back pain and neck pain. Namely, some results support a relationship between poor pulmonary function and elevated risk of back pain and neck pain, while other results do not support a relationship. These findings are generally consistent with other reviews on this topic [11,179,180].

For the intervention trials, the current review found only one RCT that assessed the efficacy of an IAQ intervention on the prevalence of back pain during the past month and no RCTs on neck pain; therefore, evidence is insufficient to make clinical recommendations. For comparison, our previous reviews did not find any RCTs assessing IAQ interventions for back pain, neck pain, or other MSDs [8,9].

The 12 RCTs uncovered in the current review assessed the efficacy of breathing exercises on pain intensity and disability related to back pain or neck pain. These RCTs were small, generally included short-term outcomes, enrolled heterogeneous groups of patients with non-specific LBP, and used a wide variety of breathing exercise types with minimal overlap across the studies. Therefore, considering the limitations and heterogeneity of the available evidence, the clinical recommendations (as shown in Table 2) about breathing exercises resulting from these RCTs should be used with caution. Conclusions from our current review differ from a previous systematic review on breathing exercises for back pain [18]. For example, the current review found moderate evidence from two RCTs, suggesting that breathing exercises alone compared to control may be useful to improve pain outcomes, and conflicting evidence from seven RCTs about the efficacy of adding breathing exercises to other interventions on pain outcomes. The previous review found evidence from seven RCTs to support the efficacy of breathing exercises to improve pain outcomes for back pain. A possible explanation for these differences is that we stratified the clinical recommendations by use of breathing exercises alone or in combination with other interventions, while the previous review did not. Further, the previous review included RCTs that we did not, such as RCTs without comparisons between groups for relevant outcomes [144], and RCTs in which the independent effects of breathing exercise could not be determined [133,134].

For neck pain, the current review found three RCTs on breathing exercises, which had mixed findings for pain, disability, and pulmonary function outcomes. For comparison, the current review and a previous review [13] found one small RCT on breathing exercises that did not support the efficacy of this intervention for pain outcomes [74].

The current review has limitations that need to be addressed in future research. For example, the combined impact of IAQ and breathing on back pain or neck pain was not assessed in any study. The available evidence was mostly from lower-level studies, and no level 1 studies (e.g., high-quality RCTs) were found, which limited assessment of causality of the observed IAQ breathing parameters, back pain, and neck pain relationships. Several pairwise comparisons had minimal studies to formulate empirical evidence statements or the grade of evidence statements. Comparisons among the studies were challenging, and meta-analysis was not possible because of the previously described limitations of the available evidence. In addition, the studies did not assess the interrelationships of numerous factors that may affect back pain and neck pain development, recovery, and prognosis, such as those reflecting what is put into the building (e.g., ergonomics, biopsychosocial factors) rather than the building itself [8,9].

The RCTs on breathing exercises uncovered in the current review had additional limitations that negatively impact generalizability. For example, the RCTs also enrolled people with heterogeneous types of back pain and neck pain without considering precise diagnostic or treatment classifications. Nearly all (11/12) of the RCTs only assessed short-term outcomes. The breathing exercises delivered among the RCTs were heterogenous and unstandardized. The RCTs also did not report if the participants achieved proper breathing patterns through the administered exercises. Moreover, none of the RCTs analyzed the relationships among breathing parameters, exercise adherence, and clinical outcomes (e.g., pain, disability). Finally, none of the RCTs assessed implementation factors, such as those described for the reach, effectiveness, adoption, implementation, and maintenance (RE-AIM) and consolidated framework for implementation research (CFIR) models [181].

A full examination of causality about the relationships of IAQ and breathing parameters with back pain and neck pain using Hill's criteria [182] was not possible since the studies found in the current review were generally lower level (i.e., no level 1 studies were uncovered). Regardless, it is biologically plausible that addressing IAQ and breathing parameters could be useful to mitigate risk factors for back pain and neck pain, as mentioned in the introduction of this manuscript. Thus, this review, along with our other reviews [8,9], provides a comprehensive initial framework on this topic that can be used to inform future research and implementation initiatives. While the available evidence from the RCTs was generally inconclusive, the combined body of evidence from the observational studies and RCTs can be used to create awareness among the diverse groups impacted by the human-building-environment interface involving IAQ, breathing, and back and neck pain.

Assuming positive findings in subsequent research, various stakeholders may benefit from the implementation of strategies to mitigate IAQ and breathing parameter risk factors related to back pain and neck pain. For healthcare stakeholders, these strategies, especially if combined with established interventions, may improve the recovery, function, quality of life, and performance of people suffering from back pain and neck pain. For occupational stakeholders, these multi-modal interventions could enhance employee productivity and reduce lost work time [183,184]. For real estate professionals, enhancements to IAQ and other HBDs could result in monetary benefits [39,185], higher tenant satisfaction and retention [8,9], and lower risk of liability related to injury or poor health [8,9]. For policymakers, the implementation of client-centered practices and policies to improve indoor environmental quality related to IAQ and breathing parameters could be influential in attenuating the global burden of human disability [8,9].

## Conclusions

Back pain and neck pain are major global burdens on individual sufferers and society. Numerous biopsychosocial factors affect the development, recovery, and prognosis of these disorders. Moreover, various interventions are available to combat their adverse consequences - many of which have modest short-term outcomes. Gaps in knowledge exist about the relationship of IAQ and breathing parameters (pulmonary function, respiratory disorders) on back pain and neck pain. This review systematically examined the peer-reviewed literature about the impact of IAQ and breathing parameters on back pain and neck pain. This search found 67 eligible studies (54 observational studies and 13 RCTs) that enrolled 345,832 participants. Key findings were the following: (1) None of the uncovered studies assessed the combined impact of IAQ and breathing parameters on back pain or neck pain. (2) Evidence indicates that poor IAQ and the presence of respiratory disorders are related to an increased risk of back pain and neck pain. (3) Conflicting evidence exists about the association between pulmonary function with back pain and neck pain. (4) Evidence for breathing exercise interventions is mixed with numerous limitations, which precludes making strong recommendations for or against their use for reducing pain and disability related to back pain and neck pain.

Overall, no level 1 studies were found, which precludes making strong statements about causality and strong recommendations about the efficacy of IAQ and breathing exercise interventions for reducing pain and disability related to back pain and neck pain. Regardless, the uncovered studies provided preliminary evidence on the relationships of IAQ and breathing parameters with back pain and neck pain, which can be used to guide future research and clinical implementation efforts. Assuming positive findings in subsequent research, a wide range of stakeholders involved with this complex human-building-environment interface can be equipped to address IAQ and breathing parameters along with other established risk factors to help those suffering from back pain and neck pain.

## Appendices

**Table 3:** Supplemental table: PubMed search strategy

Search Number	Search Details
27	22 AND 26
26	23 OR 24 OR 25
25	13 OR 14 OR 15 OR 16 OR 17 OR 18 OR 19 OR 20 OR 21
24	10 OR 11 OR 12
23	5 OR 6 OR 7 OR 8 OR 9
22	1 OR 2 OR 3 OR 4
21	"breathing exercises"[MeSH Terms] OR "breathing exercises"[All Fields] OR "breathing exercise"[All Fields] OR "breathing rehabilitation"[All Fields] OR "breathing therapy"[All Fields] OR "respiratory therapy"[All Fields]
20	"carbon dioxide sensitivity"[All Fields] OR "carbon dioxide sensitivities"[All Fields] OR "carbon dioxide hypersensitivity"[All Fields]
19	"apnea"[MeSH Terms] OR "apnea"[All Fields]
18	"dyspnea"[MeSH Terms] OR "dyspnea"[All Fields] OR "shortness of breath"[All Fields] OR "breath shortness"[All Fields] OR "breathlessness"[All Fields]
17	"respiration disorders"[MeSH Terms] OR "respiration disorders"[All Fields] OR "respiration disorder"[All Fields] OR "respiratory disorders"[All Fields] OR "respiratory disorder"[All Fields] OR "respiratory insufficiency"[MeSH Terms] OR "respiratory insufficiency"[All Fields] OR "disordered breathing"[All Fields] OR "breathing disorders"[All Fields] OR "breathing disorder"[All Fields] OR "dysfunctional breathing"[All Fields] OR "breathing dysfunction"[All Fields] OR "abnormal breathing"[All Fields] OR "over breathing"[All Fields] OR "hyperventilation"[All Fields] OR "lung diseases"[MeSH Terms] OR "lung diseases"[All Fields] OR "lung disease"[All Fields]
16	"oxygen consumption"[MeSH Terms] OR "oxygen consumption"[All Fields] OR "aerobic capacity"[All Fields]
15	"mouth breathing"[MeSH Terms] OR "mouth breathing"[All Fields] OR "nose breathing"[All Fields] OR "chest breathing"[All Fields] OR "diaphragmatic breathing"[All Fields]
14	"respiratory mechanics"[MeSH Terms] OR "respiratory mechanics"[All Fields] OR "breathing mechanics"[All Fields] OR "respiration methods"[All Fields] OR "respiration method"[All Fields] OR "breathing methods"[All Fields] OR "breathing method"[All Fields] OR "breathing techniques"[All Fields] OR "breathing technique"[All Fields]
13	"respiration"[MeSH Terms] OR "respiration"[All Fields] OR "breathing"[All Fields] OR "breathe"[All Fields] OR "breath"[All Fields]
12	"ventilation"[MeSH Terms] OR "ventilation"[All Fields] OR "ventilations"[All Fields] OR "ventilate"[All Fields]
11	"tobacco smoke pollution"[MeSH Terms] OR "tobacco smoke pollution"[All Fields] OR "second hand smoke"[All Fields] OR "environmental tobacco smoke"[All Fields]
10	"air pollution"[MeSH Terms] OR "air pollution"[All Fields] OR "air quality"[All Fields] OR "air pollution, indoor"[MeSH Terms] OR "indoor air quality"[All Fields]
9	"built environment"[MeSH Terms] OR "built environment"[All Fields]
8	"environmental illness"[MeSH Terms] OR "environmental illness"[All Fields] OR "environmental illnesses"[All Fields]
7	"indoor environmental quality"[All Fields] OR "indoor environment"[All Fields]
6	"sick building syndrome"[MeSH Terms] OR "sick building syndrome"[All Fields]
5	"healthy buildings"[All Fields] OR "healthy building"[All Fields]
4	"sciatica"[MeSH Terms] OR "sciatica"[All Fields] OR "sciaticas"[All Fields]
3	"radiculopathy"[MeSH Terms] OR "radiculopathy"[All Fields] OR "radiculopathies"[All Fields]
2	"neck pain"[MeSH Terms] OR "neck pain"[All Fields]
1	"back pain"[MeSH Terms] OR "back pain"[All Fields]

**Table 4:** Supplemental table: Characteristics and outcomes of included observational studies General - ANCOVA: Analysis of Covariance; ANOVA: Analysis of Variance; BMI: Body Mass Index; CI: Confidence Interval; COPD: Chronic Obstructive Pulmonary Disease; CT: Computed Tomography; ETS: Environmental Tobacco Smoke; F: Female; Hr: Hour; IAQ: Indoor Air Quality; M: Male; MANOVA: Multivariate Analysis of Variance; Mo: Month; MSD: Musculoskeletal Disorder; MSK: Musculoskeletal; NA: Not Applicable; NDI: Neck Disability Index; NMQ: Nordic Musculoskeletal Questionnaire (original or modified); NPRS: Numerical Pain Rating Scale; NR: Not Reported; NRS: Numerical Rating Scale; NS: Not Significant (p > 0.05); ODI: Oswestry Disability Index (original or modified); OR: Odds Ratio; PR: Prevalence Ratio; PRO: Patient Reported Outcome; RMDQ: Roland Morris Disability Questionnaire; RPE: Rating of Perceived Exertion; RR: Relative Risk; VAS: Visual Analog Scale; Wk: Week; Y: Year. Pulmonary function tests - BHT: Breath Hold Time; ERV: Expiratory Reserve Volume; ERV: Expiratory Reserve Volume; ETCO2: End-Tidal CO2; FEF: Forced Expiratory Flow; FEF25-75: Forced Mid Expiratory Flow; FEV/VC ratio: Ratio of Forced Expiratory Volume to Vital Capacity; FEV/FVC ratio: Ratio of Forced Expiratory Volume to Forced Vital Capacity; FEV: Forced Expiratory Volume; FEV0.5: Forced Expiratory Volume in 0.5 sec; FEV1.0: Forced Expiratory Volume in 1 sec; FEVC: Forced Expiratory Vital Capacity; FIV0.5: Forced Inspiratory Volume in 0.5 sec; FIV1.0: Forced Inspiratory Volume in 1 sec; FIVC: Forced Inspiratory Vital Capacity; FVC: Forced Vital Capacity; IC: Inspiratory Capacity; MEP: Maximum Expiratory Pressure; MIP/MEP ratio: Ratio of Maximum Inspiratory Pressure to Maximum Expiratory Pressure; MIP: Maximum Inspiratory Pressure; MV: Minute Volume; MVV: Maximum Voluntary Ventilation; PEF: Peak Expiratory Flow; PEFR: Peak Expiratory Flow Rate; PETCO2: Partial Pressure of End-Tidal CO2; PFT: Pulmonary Function Test; PIF: Peak Inspiratory Flow; RR: Respiration Rate; TV: Tidal Volume; VC: Vital Capacity.

Author, Year, Country, Funding Source	Population Studied, Sample Size (Gender), Age, Case, Control	Eligibility Criteria	Outcomes Assessed: (1) Back Pain, Neck Pain; (2) IAQ, Pulmonary Function, Respiratory Disorder	Analysis. Results
Angst et al. [49], 2017, Switzerland, NR	General population from Zurich, Switzerland, 499 (Gender: NR), 27-50 y, NA, NA	Inclusion: General population from Zurich, Switzerland with data available within Zurich Cohort Study, completed interviews from 1986-2008. Exclusion: NR.	(1) Back Pain, Neck Pain: Incidence, prevalence, and intensity of low back pain and neck pain over the past 12 mon assessed via interview and VAS (PRO). (2) Respiratory Disorder: Prevalence and incidence of asthma assessed via interview: yes/no (PRO).	Logistic regression. Asthma was significantly associated with an increased risk of low back pain and neck pain over the past 12 mo (low back pain: OR 5.76, 95% CI 4.05-8.19, p < 0.05. neck pain: OR 2.42, 95% CI 1.82-3.22, p < 0.05).
Astrand [51], 1987, Sweden, Swedish Environment Fund	Manual and office workers in paper mill, 391 (0 F, 391 M), 45-55 y (median), NA, NA	Inclusion: Manual workers and office workers at 4 sites of Svenska Cellulosa AB paper mill, male, age 35-65 y. Exclusion: NR.	(1) Back Pain: Back pain defined as "Do you often have lumbago or pain in your back": yes/no (PRO). Normal or abnormal back defined from an aggregate variable of clinical signs and symptoms: (yes/no). (2) Respiratory Disorder: Shortness of breath defined as "Do you get out of breath going upstairs": yes/no (PRO).	Logistic regression. The risk of shortness of breath going upstairs was significantly greater in those with abnormal backs compared to normal (OR 2.1, p = 0.01). No significant difference in shortness of breath between those with and without back pain.
Awadallah et al. [52], 2021, Egypt, None	Adults with and without neck pain, 150 (22 F, 138 M), 27.2 ± 68.2 y, Case (n = 75): Neck pain > 6 mo duration, Control (n = 75): No neck pain	Inclusion: Age ≥ 18 y, neck pain > 6 mo. Exclusion: arm pain or structural malformations related to neck pain, clinical abnormalities, history of respiratory disease, MSK disease, tumor, infection, spinal fractures, spinal cord compression that required surgery, or spine surgery current or past smokers, BMI >30 or ˂18.5. Controls (without neck pain) were matched to cases on NR.	(1) Neck Pain: Pain intensity assessed with VAS (PRO). Disability assessed with NDI (PRO). (2) Pulmonary function assessed with spirometer: FVC, FEV1.0, FEF25-75, PEFR, FEV1.0/FVC.	T-test. FVC, FEV1.0, FEV1.0/FVC, FEF25-75, and PEFR were significantly worse in those with chronic neck pain compared to asymptomatic controls (mean difference - FVC: 16.54, p < 0.001. FEV1.0: 3.50, p = 0.03. FEV1.0/FVC: -18.65, p < 0.001. FEF25-75: 15.47, p = 0.003. PEFR: 16.08, p < 0.001).
Beynon et al. [53], 2021, Australia, Various university and foundation funding sources	General population from Australia, 1,235 (640 F, 595 M), 22.2 ± 0.8 y, NA, NA	Inclusion: General population from Australia with 22-y follow-up data available within Raine Study Gen2. Exclusion: NR.	(1) Back Pain: Impactful back pain, defined as "Yes" answer to ≥ 1 item on a 4-5 item questionnaire about back pain: yes/no (PRO). (2) Respiratory Disorder: Health questionnaire with exposure categories on chronic inflammatory conditions (e.g., respiratory conditions) completed by participant or parent (PRO).	Logistic regression. At 22-y follow-up, respiratory conditions were significantly associated with an increased risk of developing future low back pain (OR 1.29, 95% CI 1.07-1.54, p < 0.05).
Borisut et al. [54], 2021, Thailand, Chulalongkorn University	Female office workers with and without neck pain, 40 (40 F, 0 M), 29.9 ± 3.4 y, Case (n = 20): Neck pain > 6 mo duration, Control (n = 20): No neck pain	Inclusion: Female, age 20-45 y, work-related neck pain > 6 mo, computer use at work ≥ 4 hr/d. Baseline neck pain > 30 mm on 0-100mm VAS. Exclusion: Non-MSK neck or shoulder pain, neurological signs, spinal or chest surgery history, smoking history, and pregnant. Controls (without neck pain) were matched to cases of age and BMI.	(1) Neck Pain: Pain intensity assessed with VAS (PRO). (2) Pulmonary function assessed with spirometer: MIP, MEP.	T-test. MIP and MEP were significantly worse in those with chronic neck pain compared to asymptomatic controls (mean difference - MIP: - 13.14, p = 0.001. MEP: -10.30, p = 0.001).
Cagnie et al. [56], 2007, Belgium, NR	Office workers, 512 (225 F, 287 M), 30-39 y (median), NA, NA	Inclusion: Ofﬁce computer workers from 10 companies in Belgium. Exclusion: NR.	(1 Neck Pain: Neck pain in past 12 mo: 4-point Likert scale (PRO) 2. IAQ: Exposure to lack of fresh air, dry air, and stench at work: yes/no (PRO).	Logistic regression. Experiencing dry air was significantly associated with an increased risk of neck pain in the past 12 mo: OR 1.94, 95% CI 1.28-2.70, p = 0.001. Fresh air, stench: NR.
Carnow [57], 1981, Canada, NR	Smelting factory workers, 1,242 (gender: NR), 31-40 y (median), NA, NA	Inclusion: Hourly employees of smelting factory. Exclusion: On disability leave, worked at smelting factory for ≤ 3 mo.	(1) Back Pain, Neck Pain: MSK disorder history while at current job: yes/no (PRO); MSK symptoms frequency: 0-15, categorized as low or high frequency (PRO); Back or neck surgery history: yes/no (PRO). (2) IAQ: Fluoride exposure in ambient air at work: exposure risk index (low, medium, high).	Chi-square. High exposure to ambient air fluoride (compared to low exposure) was significantly associated with an increased prevalence of a history of back pain, neck pain, and related MSK disorders: Chi-square = 42.9, p < 0.001. High exposure to ambient air fluoride (compared to low exposure) was significantly associated with an increased prevalence of a history of back or neck surgery: Chi-square = 10.62, p < 0.005. No significant relationship between high exposure to ambient air fluoride (compared to low exposure) and current back pain, neck pain, and related MSK disorders.
Cheon et al., 2020, Korea, NR	Adults with and without neck pain, 78 (40 F, 38 M), 50.9 ± 8.4 y, Case (n = 48): Neck pain ≥ 6 mo duration, Control (n = 30): No neck pain past 6 mo	Inclusion: Neck pain ≥ 6 mo for ≥ 1 d/wk, age 19-65 y. Exclusion: History of surgery or trauma in the head, neck trunk, limbs, myopathy, congenital anomalies in the spine, COPD, asthma, obesity, smoking, autoimmune disease, abnormal chest XR. Controls (without neck pain for the past 6 mo) were matched to cases on NR.	(1) Neck Pain: Disability assessed with NDI (PRO). (2) Pulmonary function assessed with spirometer: MIP, MEP.	T-test. In females, MIP and MEP were significantly worse in those with chronic neck pain compared to asymptomatic controls (MIP: case 76.40 ± 7.76, control 85.86 ± 8.49, p = 0.002. MEP: case 92.44 ± 9.52, control 104.26 ± 7.85, p < 0.001). In males, MEP was significantly worse in those with chronic neck pain compared to asymptomatic controls (case 119.21 ± 6.57, control 126.13 ± 13.08, p = 0.04). No significant difference between those with chronic neck pain and asymptomatic controls in MIP.
Chuang et al. [59], 2008, China, Astellas Pharma	Residents of Taiwan, 4,011 (2,377 F, 1,634 M), median 31-50 y, NA, NA	Inclusion: Residents of Taiwan, age ≥ 15 y, contact information available in Taiwan phonebook. Exclusion: Language barrier.	(1) Back Pain: Prevalence of back pain assessed via interview: yes/no (PRO). (2) Respiratory Disorder: Prevalence of snoring or sleep apnea assessed via interview: yes/no (PRO).	Chi-square. Snoring and sleep apnea were significantly associated with an increased risk of low back pain (snoring: yes 33.7%, no 25.0%, p < 0.001. sleep apnea: yes 48.5%, no 28.1%, p < 0.001).
Clark et al. [60], 2014, United States, National Institutes of Health, Veterans Administration, American Federation for Aging Research	Participants in cohort 12 of the Medicare Health Outcomes Survey, 113,432 (64,656 F, 48,776 M) age < 75: 63%, ≥ 75 y: 37%, NA, NA	Inclusion: Completed follow-up survey as a participant in cohort 12 of the Medicare Health Outcomes Survey. Exclusion: NR.	(1) Back Pain: Low back pain in the past 4 weeks that interfered with usual activities: yes/no (PRO). (2) Respiratory Disorder: Dyspnea in the past 4 weeks while sitting, resting, walking, or climbing stairs: yes/ no (PRO).	T-test, logistic regression. At 2-y follow-up, the prevalence of back pain was significantly higher in those with dyspnea compared to those without (dyspnea 49%, no dyspnea 12%, p < 0.001). The risk of reporting dyspnea was significantly greater in those with back pain compared to those without back pain (RR 1.76, 95% CI 1.71-1.82).
Dağ et al. [61], 2022, Turkey, No extramural funding	Adults with and without neck pain, 52 (25 F, 27 M), 26.4 ± 7.5 y, Case (n = 25): Neck pain ≥ 6 mo duration, Control (n = 27): No neck pain	Inclusion: Age 18-42 y, neck pain ≥ 6 mo, NDI ≥ 15. Exclusion: Disc herniation with neurologic deﬁcits, abnormalities of thoracic cage or spine region, history of cervical or thoracic trauma or malignancy, systemic disorder, serious comorbidity that could impact pulmonary function tests. Controls (without neck pain over the past 12 mo) were matched to cases on age and gender.	(1) Neck Pain: Disability assessed with NDI (PRO). (2) Pulmonary function assessed with spirometer: FEV1.0, FVC, FEV1.0/FVC, FEF25-75, PEF, MVV.	T-test. FEV1.0, FVC, FEF25-75, and MVV were significantly worse in those with chronic neck pain compared to asymptomatic controls (FEV1.0 - case 90.84 ± 14.11, control 99.37 ± 10.26, p = 0.015. FVC - case 95.76 ± 12.66, control 102.96 ± 10.33, p = 0.029. FEF25-75 - case 75.88 ± 23.51, control 87.78 ± 16.89, p = 0.04. MVV - case 82.80 ± 17.41, control 93.22 ± 18.44, p = 0.42). No significant difference between those with chronic neck pain and asymptomatic controls in FEV1.0/FVC and PEF.
de Luca et al. [63], 2017, Australia, Various private institute, foundation, and scholarship funding sources	Community-dwelling older women, 579 (579 F, 0 M), 64.6 ± 1.5 y, NA, NA	Inclusion: Community-dwelling Australian resident, female, a participant in the Australian Longitudinal Study on Women’s Health, age 61-66 y, yes answer to the question "In the past 3 years have you been diagnosed or treated for arthritis/rheumatism.” Exclusion: NR.	(1) Back Pain, Neck Pain: Prevalence of spinal pain in the past mo assessed by questionnaire: yes/no (PRO). (2) Respiratory Disorder: Prevalence of pulmonary disease in past 3 y assessed by questionnaire: yes/no (PRO).	Logistic regression. Pulmonary disease was significantly associated with an increased risk of back pain or neck pain over the past mo (OR 1.66, 95% CI 1.04-2.65, p = 0.034).
de Miguel-Díez et al. [64], 2018, Spain, NR	Non-institutionalized adults with and without COPD, 4,502 (2,404 F, 2,098 M), median 60-79 y, Case (n = 2,251): COPD, Control (n = 2,251): No COPD	Inclusion: Non-institutionalized residents of Spain, participants in European Health Interview Surveys for Spain (EHSS), diagnosis of COPD, age ≥ 35 y. Exclusion: NR. Controls (without COPD) were matched to cases on age, gender, and residence.	(1) Back Pain, Neck Pain: Prevalence of chronic low back pain and chronic neck pain in the past 12 mo assessed by questionnaire: yes/no (PRO). 2. Respiratory Disorder: Prevalence of COPD assessed by questionnaire (PRO).	Logistic regression. COPD was associated with an increased risk of chronic low back pain and chronic neck pain over the past 12 mo (low back pain: OR 1.38, 95% CI 1.16-1.64, p < 0.05) (neck pain: OR 1.21, 95% CI 1.02-1.45, p < 0.05).
Dewey et al. [65], 1989, United Kingdom, Health and Safety Executive	College, fire, ambulance, and hospital service workers, and Colleges, 909 (317 F, 592 M), age NR, NA, NA	Employees of Fire, Ambulance, and Hospital Services, or universities and colleges in NW England and N Wales. Exclusion: NR.	(1) Back Pain: Prevalence and incidence of low back pain assessed via questionnaire with 6 categories at 12-mo follow-up (PRO). (2) Pulmonary function assessed with spirometer: FEVC, FEV0.5, FEV1.0, PEF, FIVC, FIV0.5, FIV1.0, PIF. Chest symptoms were assessed by Medical Research Council Questionnaire (PRO).	ANOVA, log-linear model, chi-square. At 12-mo follow-up: In females, FEVC was significantly worse in those with low back pain compared to those without low back pain (chronic low back pain 3.36, no low back pain 3.53, p < 0.05). No significant difference between those with chronic low back pain and those without low back pain in FEV0.5, FEV1.0, PEF, FIVC, FIV0.5, FIV1.0, PIF. In males, FEVC, FEV0.5, and FEV1.0 were significantly worse in those with low back pain compared to those without low back pain (FEVC: chronic low back pain 4.87, no low back pain 5.19, p < 0.05. FEV0.5: chronic low back pain 2.94, no low back pain 3.14, p = 0.05. FEV1.0: chronic low back pain 3.92, no low back pain 4.24, p = 0.05). No significant difference between those with chronic low back pain and those without low back pain in PEF, FIVC, FIV0.5, FIV1.0, PIF. Chest symptoms were significantly associated with an increased risk of low back pain (chi-square 29.4, p < 0.01).
Dimitriadis et al. [67], 2013, Greece, NR	Adults with and without neck pain, 90 (64 F, 26 M), 35.7 ± 14.3 y, Case (n = 45): Chronic neck pain, Control (n = 45): No neck pain	Inclusion: Individuals with neck pain (cases) and without neck pain (control). Cases: neck pain ≥ 6 mo, symptoms ≥ 1 X/wk, ages 18-65 y. Controls (without back pain): matched to cases on gender, age, height, and weight. Exclusion: Spinal or chest surgery, smoking history, traumatic neck injury. MSK pain in other body regions, BMI > 40, clinical abnormalities of thoracic cage or spine, occupational exposures, serious comorbidities, diabetes, malignancy. Controls (without neck pain) were matched to cases on age, gender, height, and weight.	(1) Neck Pain: Prevalence of neck pain to determine study eligibility (PRO). (2) Pulmonary function assessed with spirometer: MIP, MEP, MIP/MEP.	T-test. MIP and MEP were significantly worse in those with chronic neck pain compared to healthy controls (mean difference - MIP: -13.9, 95% CI -25.9 to -2.7, p < 0.05. MEP: -19.5, 95% CI -33.6 to -5.5, p < 0.01). No significant difference between those with chronic neck pain and asymptomatic controls in MIP/MEP.
Dimitriadis, et al. [68], 2014, Greece, NR	Adults with and without neck pain, 90 (64 F, 26 M), 35.7 ± 14.3 y, Case (n = 45): Chronic neck pain, Control (n = 45): No neck pain	Inclusion: Individuals with neck pain (cases) and without neck pain (control). Cases: neck pain ≥ 6 mo, symptoms ≥ 1 X/wk, ages 18-65 y. Controls (without back pain): matched to cases on gender, age, height, and weight. Exclusion: Spinal or chest surgery, smoking history, traumatic neck injury. MSK pain in other body regions, BMI > 40, clinical abnormalities of thoracic cage or spine, occupational exposures, serious comorbidities, diabetes, malignancy. Controls (without neck pain) were matched to cases on age, gender, height, and weight.	(1) Neck Pain: Prevalence of neck pain to determine study eligibility (PRO). (2) Pulmonary function assessed with spirometer: VC, FVC, IC, ERV, FEV, FEF, PEF, and MVV.	T-test. VC, ERV, FVC, and MVV were significantly worse in those with chronic neck pain compared to healthy controls (VC: mean difference -7.4, 95% CI -13.6 - -1.1, p < 0.05. ERV: mean difference -12.6, 95% CI -25.1 - 0, p < 0.05. FVC: mean difference -6.5, 95% CI -12.6 - -0.5, p < 0.05. MVV mean difference -12.2, 95% CI -21.2 - -3.2, p < 0.01). No significant difference between those with chronic neck pain and asymptomatic controls in IC, FEV, FEF, and PEF.
Eriksen [69], 2004, Norway, Norwegian Research Council	Nurses' aides, 4,744 (4,558 F, 186 M), 45-49 y (median), NA, NA	Inclusion: Certified nurses’ aides from the Norwegian Union of Health and Social Workers. Exclusion: On leave at baseline.	(1) Back Pain, Neck Pain: Sick leave related to back or neck pain > 14 days during the past 12 mo (PRO). (2) IAQ: ETS exposure during childhood: no/sometimes/often (PRO).	Logistic regression. Childhood ETS exposure (sometimes or often) was significantly associated with increased risk of sick leave > 14 days related to neck pain, upper back pain, and low back pain during subsequent 12 mo (neck pain: OR 1.34, 95% CI 1.04-1.73, p < 0.05) (upper back pain: OR 1.49, 95% CI 1.07-2.06, p < 0.05) (low back pain: OR 1.21, 95% CI 0.97-1.50, p = 0.09).
Fahad et al. [70], 2020, Iraq, No extramural funding	Adults with and without cervical spinal stenosis, 100 (60 F, 40 M), 49.9 ± 11.7 y, Case (n = 40): Cervical spinal stenosis, Control (n = 60): No cervical spinal stenosis	Inclusion: Cervical spinal stenosis of 1-24 mo duration. Exclusion: Obesity, smoking, COPD, malignancy, congenital spinal stenosis, peripheral neuropathy, diabetes mellitus, metabolic diseases, and psychological disorders. Controls (without cervical spinal stenosis) were matched to cases on NR.	(1) Neck Pain: Radiographic assessment (CT, plain film) of cervical spinal stenosis. (2) Pulmonary function assessed with spirometer: VC-inspiration, VC-expiration, FEV1.0, FVC, PEF, MVV, FEV1.0/FVC.	T-test. VC-expiration, FEV1.0, FVC, PEF, and MVV were significantly worse in those with cervical spinal stenosis compared to asymptomatic controls (VC-expiration - case 79.23 ± 11.24, control 90.86 ± 11.80, p < 0.001) (FEV1.0 - case 89.16 ± 15.59, control 105.53 ± 14.04, p < 0.001) (FVC - case 77.26 ± 13.10, control 90.56 ± 12.69, p < 0.001) (PEF - case 85.21 ± 14.82, control 93.21 ± 21.32, p = 0.42) (MVV - case 98.42 ± 16.77, control 106.14 ± 18.55, p = 0.037). No significant difference between those with cervical spinal stenosis and asymptomatic controls in VC-inspiration and FEV1.0/FVC.
Fuentes-Alonso et al. [71], 2020, Spain, SEPAR, NEUMOMADRID, FIS - Health Research Fund, FEDER	Community-dwelling adults with and without COPD, 2,068 (gender: NR), ≥ 35 y (inclusion criteria), Case (n = 1,034): COPD, Control (n = 1,034): No COPD	Inclusion: Data available within Spanish National Health Survey 2017, age ≥ 35 y, reside in the main family dwelling, self-reported COPD (for cases). Exclusion: NR. Controls (without COPD) were matched to cases on age, gender, and residence.	(1) Back Pain, Neck Pain: Chronic back pain and chronic neck pain variables derived from positive responses to three health survey items on back pain and neck pain: yes/no (PRO). (2) Respiratory Disorder: COPD sufferer variable derived from positive responses to three health survey items on COPD: yes/no (PRO).	McNemar test. COPD cases had a significantly higher prevalence of chronic low back pain and chronic neck pain compared to non-COPD controls (low back pain: case 45.2%, control 28.3%, p < 0.001) (neck pain: case 38.2%, control 22.8%, p < 0.001).
Hagins et al. [73], 2011, United States, National Institutes of Health	Adults with and without low back pain, 62 (32 F, 30 M), 34.6 ± 10.2 y, Case (n = 32): Low back pain ≥ 12 mo, Control (n = 30): No low back pain in past 6 mo	Inclusion: Age 21-50 y, low back pain for ≥ 12 mo, pain intensity ≤ 2/10 during activity. Exclusion: Inability to participate in work/school activities, history of spinal surgery or any surgery past 12 mo that impacted lifting ability, respiratory conditions, pregnancy, cardiac conditions, spinal disease, or deformity. For controls, low back pain in the past 6 mo, disc herniation, radiculopathy, spinal stenosis, ≥ grade 3 spondylolisthesis, spinal tumor, infection, fracture. Controls (no low back pain) were matched to cases on age, gender.	(1) Back Pain: Pain intensity assessed with VAS (PRO). Disability assessed with ODI (PRO). (2) Pulmonary function assessed with spirometer: resting TV and VC, %VC during lifts.	ANCOVA. %VC during lifts was significantly greater in those with chronic low back pain compared to asymptomatic controls (case 48.1%, control 40.9%, p < 0.05). No significant difference between those with chronic low back pain and asymptomatic controls in resting TV and VC.
Hamaoui et al., 2002, France, NR	Adults with and without low back pain, 20 (0 F, 20 M), 32 ± 6 y, Case (n = 10): Low back pain ≥ 3 mo, Control (n = 10): No history of low back pain	Inclusion: Age 20-40 y, low back pain for ≥ 3 mo. Exclusion: Disease associated with low back pain. Controls (without a history of low back pain) were matched to cases of gender.	(1) Back Pain: NR. (2) Pulmonary function assessed with respiratory sensor: RR, MV.	ANOVA, T-test. No significant difference between those with chronic low back pain and asymptomatic controls in RR and MV.
Hestbaek et al. [76], 2006, Denmark, Foundation for Chiropractic Research and Postgraduate Education	General population from Denmark, 6,554 (3,682 F, 2,868 M), 20-30 y (at 8-y follow-up), NA, NA	Inclusion: General population from Denmark, data available in Danish Twin Register, born during 1972-1982. Exclusion: NR.	(1) Back Pain: Pain assessed with NMQ (PRO). (2) Respiratory Disorder: Lifetime prevalence of asthma assessed within health questionnaire: yes/no (PRO).	Logistic regression. Asthma in adolescence was significantly associated with an increased risk of developing future persistent low back pain (OR 1.34, 95% CI 1.10-1.62, p < 0.05).
Hurwitz et al. [77], 1999, United States, NR	Residents of United States, 6,836 (3,725 F, 3,111 M), median 30-39 y, NA, NA	Inclusion: Non-institutionalized residents of the United States, participants in the 3rd National Health and Nutrition Examination Survey. Exclusion: NR	(1) Back Pain: Prevalence of low back pain in the past 12 mo assessed via questionnaire: yes/no (PRO). (2) Respiratory Disorder: Prevalence of asthma assessed by questionnaire: yes/no (PRO).	Logistic regression. Asthma was significantly associated with an increased risk of low back pain over the past 12 mo (OR 1.56, 95% CI 1.08-2.24, p < 0.05).
Ignatius et al. [78], 1993, China, NR	Typist workers, 170 (170 F, 0 M), 31.5 ± 7.0 y, NA, NA	Inclusion: Typists working at Government Housing Department. Exclusion: NR.	(1) Back Pain, Neck Pain: MSK symptoms and fatigue point prevalence assessed via interview (PRO). (2) IAQ: Polluted indoor air: yes/no/unsure interview (PRO).	Chi-square, T-test, Logistic regression. No significant relationship between polluted indoor air with back pain or neck pain.
Kapreli et al. [79], 2009, Greece, NR	Individuals with and without neck pain, 24 (14 F, 10 M), 29.3 ± 3.3 y, Case (n = 12): Neck pain ≥ 6 mo, Control (n = 12): No neck pain	Inclusion: Neck pain ≥ 6 mo associated with cervical joint dysfunction, neck pain frequency ≥ 1X/wk. Exclusion: History of the cervical spine, abdominal, or chest surgery, a participant in a neck exercise program in the past 12 mo, tobacco use, occupational exposure, BMI > 40, COPD, malignancy, various other clinical abnormalities. Controls (without neck pain) were matched to cases on age, gender, height, weight, and physical activity level.	(1) Neck Pain: Pain intensity assessed with VAS (PRO). Disability assessed with NDI (PRO). (2) Pulmonary function assessed with spirometer: MVV, MIP, MEP, FVC, FEV1.0, FEV1.0/FVC, FEV1.0/FVC %, FEF25-75, FEF25, FEF75, FEF50, PEF, and VC.	T-test. MVV, MIP, and MEP were significantly worse in those with neck pain compared to asymptomatic controls (MVV - mean difference -19.22, p = 0.042) (MIP - mean difference -24.17, p = 0.010) (MEP - mean difference -22.83, p = 0.018). No significant difference between those with neck pain and asymptomatic controls in FVC, FEV1.0, FEV1.0/FVC, FEV1.0/FVC %, FEF25-75, FEF25, FEF75, FEF50, PEF, and VC.
Kupeli et al. [81], 2021, Turkey, NR	Patients undergoing PFT and thoracic CT scan, 165 (52 F, 113 M), 62.6 ± 9.7 y, NA, NA	Inclusion: Underwent PFT and thoracic CT scan within 2 wk of each other. Exclusion: History of spinal surgery, trauma, osteoporosis, or scoliosis, COPD flare-up past 2 mo.	(1) Back Pain: CT scans to determine costotransverse, facet, and intervertebral joint arthroscopy, back pain severity on 0-10 VAS categorized to back pain yes/no (PRO). (2) Respiratory Disorder: PFT to determine COPD.	T-test, chi-square. The prevalence of back pain, and costotransverse, facet, and intervertebral joint arthroscopy was significantly higher in those with COPD compared to those without COPD (back pain - COPD: 43.7%, No COPD: 20.9%, p < 0.001. costotransverse: - COPD: 26.5%, No COPD: 2.8%, p < 0.001. facet: - COPD: 29.6%, No COPD: 9.9%, p < 0.001. intervertebral: - COPD: 45.3%, No COPD: 29.7%, p < 0.001.)
Lambrechts, 2023 [82]. United States, NR	Patients undergoing lumbar MRI, 605 (328 F, 277 M), 53.2 ±14.2 y, NA, NA	Inclusion: Patients undergoing lumbar MRI with data available in electronic medical records. Exclusion: Unavailable MRI data, presence of scoliosis, compression fractures, spondylolisthesis, or lumbar fusion surgery.	(1) Back Pain: Lumbar disc degeneration assessed from MRI and graded with Griffith-modified Pfirrmann score. (2) Respiratory Disorder: Prevalence of asthma, COPD, and sleep apnea assessed from medical history data: yes/no (PRO).	Chi-square, Fisher's exact test. The presence of COPD was significantly associated with an increased risk of disc degeneration at the T12-L1 (p = 0.947), L1-L2 (p = 0.001), L2-L3 (p = 0.01), L4-L5 (p = 0.015), and L5-S1 levels (p = 0.024). No significant relationship between COPD and disc degeneration at L3-L4. The presence of sleep apnea was significantly associated with an increased risk of disc degeneration at the L1-L2 (p = 0.042) and L4-L5 (p = 0.006) levels. No significant relationship between sleep apnea and disc degeneration at T12-L1, L2-L3, L3-L4, and L5-S1 levels. No significant relationship between asthma and lumbar disc degeneration at any level.
Lee et al. [83], 2017, Canada, NR	Adults with and without COPD, 128 (gender: 56 F, 72 M), 69 ± 12 y, Case (n = 64): COPD, Control (n = 64): No COPD	Inclusion: The presence of COPD is defined as FEV1.0/FVC ratio < 70, smoking history > 10 pack y (for cases). Exclusion: Other concurrent respiratory diseases, malignancy, acute exacerbation of COPD, MSDs within the past 4 weeks. Controls (without COPD or MSDs) were matched to cases of age and gender.	(1) Back Pain, Neck Pain: Pain assessed with Brief Pain Inventory, Pain duration, and frequency, Extended Aberdeen Back Pain Scale (PRO). (2) Respiratory Disorder: Pulmonary function assessed with spirometer: COPD defined as FEV1.0/FVC ratio <70.	T-test, Mann-Whitney U test, Chi-square. COPD cases had significantly higher back pain intensity compared to non-COPD controls (case: 26.3 ± 22.6, control: 12.8 ± 10.6, p = 0.003). COPD cases had a significantly higher prevalence of upper back pain compared to non-COPD controls (case: 25%, control: 7%, p < 0.05). No significant difference in the prevalence of low back pain and neck pain between COPD cases and non-COPD controls.
Li et al. [84], 2017, Australia	Adults, 360 (0 F, 360 M), 60.0 ± 11.1 y, NA, NA	Inclusion: Community-dwelling males in Australia, participants in the Men Androgen Inflammation Lifestyle Environment and Stress study. Exclusion: NR.	(1) Back Pain: Prevalence of back pain over the past mo assessed via questionnaire: yes/no (PRO). (2) Respiratory Disorder: Obstructive sleep apnea assessed with in-home polysomnography.	No significant relationship between obstructive sleep apnea and low back pain.
López-de-Uralde-Villanueva et al. [85], 2018, Spain, NR	Adults with and without neck pain, 75 (NR), 39.9 ± 13.9 y, Case (n = 44): Neck pain > 3 mo, Control (n = 31): No neck pain past 12 mo	Inclusion: Age 18-65 y, neck pain > 3 mo assessed by VAS. Exclusion: History of cervical trauma or surgery, neuropathic pain, inflammatory lesion in the neck, injury-related neck pain. Controls (without neck pain in the past 12 mo) were matched to cases on NR.	(1) Neck Pain: Pain intensity assessed with VAS (PRO). Disability assessed with NDI (PRO). (2) Pulmonary function assessed with spirometer: MIP, MEP, FEV1.0, FVC, FEV1.0/FVC, and PEF.	T-test. MIP, MEP, FEV1, FVC, PEF were significantly worse in those with neck pain compared to those without (mean difference, 95% CI) (MIP: 12.39, 1.76-23.02, p < 0.05) (MEP: 22.84, 8.17-37.52, p < 0.05) (FEV1: 0.56, 0.23-0.90, p < 0.05) (FVC: 0.59, 0.15-1.02, p < 0.05) (PEF: 1.44, 0.50-2.38, p < 0.05). No significant difference between those with chronic neck pain and asymptomatic controls in FEV1/FVC.
Lu [86], 2008, Philippines, NR	Export processing zone workers, 444 (444 F, 0 M), age: NR, NA, NA	Inclusion: Export processing zone workers from 24 manufacturing companies in the Philippines, female. Exclusion: NR.	(1) Back Pain: Back pain prevalence: yes/no (PRO). (2) IAQ: Exposure to indoor chemicals, dust, and poor ventilation at work assessed via questionnaires and interviews: yes/no (PRO).	Logistic regression. No significant relationships of exposure to indoor chemicals, dust, and poor ventilation at work with back pain.
Lunardi et al. [87], 2011, Brazil, NR	Adults with and without asthma, 45 (32 F, 13 M), 21-55 y (range), Case (n = 30): Asthma, Control (n = 15): No asthma, respiratory, or allergic diseases	Inclusion: The presence of asthma defined by Global Initiative for Asthma criteria. Exclusion: Sports or physical activities in the past 6 mo, systemic or rheumatologic diseases, history of MSK injury or trauma. Controls (without asthma, respiratory, or allergic diseases) were matched to cases on age, gender, and BMI.	(1) Back Pain, Neck Pain: Chronic neck, shoulder, and lower thoracic pain determined by interview (PRO). (2) Respiratory Disorder: Asthma determined by Global Initiative for Asthma criteria and categorized as mild and severe persistent asthma.	ANOVA. Prevalence of chronic neck, shoulder, and lower thoracic pain was significantly higher in those with asthma compared to those without asthma (asthma: 46.6%, no asthma: 0%, p < 0.05).
Magnavita et al. [88], 2011, Italy, NR	Hospital workers, 1,744 (977 F, 767 M), 44.9 ± 8.9 y, NA, NA	Inclusion: Workers from 3 hospitals in the Lazio region of Italy. Exclusion: NR.	(1) Back Pain: MSK pain in past 12 mo: NMQ (PRO). (2) IAQ: Environmental section of IAQ/MM-040 questionnaire with items on "Have you been annoyed in the last 3 mo by any of these factors in the workplace?" Categorized for other environmental complaints (stuffy air, dry air, unpleasant smells, static electricity, passive smoke, dust): no, sometimes, often every week (PRO).	Logistic regression. Other environmental complaints (air, dust) were significantly associated with increased risk of low back pain (OR 2.18, 95% CI 1.75-2.71, p < 0.05).
Mohan et al. [90], 2018, Malaysia, NR	Adults with and without low back pain, 68 (66 F, 2 M), 23.0 ± 1.6 y, Case (n = 34): Low back pain ≥ 6 mo, Control (n = 34): No low back pain over past 12 mo	Inclusion: Age 18-55 y, low back pain ≥ 6 mo, ≥ 3 episodes of low back pain over past 6 mo, pain intensity 2-5/10, FEV/FVC > 80%. Exclusion: Chronic respiratory disease, history of spinal surgery, pregnancy, current light smokers, smoking pack history of > 5. Controls (without low back pain over the past 12 mo) were matched to cases on gender.	(1) Back Pain: Pain intensity assessed with NRS (PRO). (2) Pulmonary function assessed with spirometer: MVV, MIP, and MEP.	T-test, Mann-Whitney U test. No significant difference between those with chronic low back pain and asymptomatic controls in MVV, MIP, and MEP.
Noormohammadpour et al. [81], 2017, Iran, Tehran University of Medical Sciences & Health Services, Centre for Disease Control and Management	General population, 7,889 (4,745 F, 3,144 M), 50.9 ± 13.1 y, NA, NA	Inclusion: Resident of Iran, a household in 429 districts as identified by postal code. Exclusion: NR.	(1) Back Pain, Neck Pain: Chronic low back pain and neck pain > 3 mo over the past y assessed with a questionnaire (PRO). (2) IAQ: ETS exposure assessed via interview (PRO).	Chi-square, Logistic regression. Exposure to ETS (passive smoking) was associated with increased risk of chronic low back pain and chronic neck pain (low back pain: OR 1.26, 95% CI 1.11-1.43, p < 0.05) (neck pain OR 1.31, 95% CI 1.12-1.54, p < 0.05).
O'Sullivan et al. [93], 2002, Australia, NR	Individuals with and without sacroiliac joint pain, 26 (22 F, 4 M), 31.9 ± 11.3 y, Case (n = 13): Sacroiliac joint pain ≥ 3 mo, Control (n = 13): No low back pain over past 12 mo	Inclusion: Sacroiliac joint pain > 3 mo, positive active straight leg raise, ≥ 4 positive sacroiliac joint provocation tests. Exclusion: Neurological dysfunction, facial pain that precludes masking, significant respiratory disease history, pregnancy, < 6 mo postpartum, BMI < 31 kg/m2. For control: inability to perform active straight leg raise, history of low back or lower extremity disorder in past 6 mo, lumbar, spine, pelvis, chest or abdomen surgery in past 12 mo, inflammatory disorder. Controls (without low back pain over the past 12 mo) were matched to cases on age, BMI, and gender.	(1) Back Pain: Clinical assessment. (2) Pulmonary function assessed with spirometer: MV, RR, TV.	ANOVA. MV and RR were significantly higher (during active straight leg raise) in those with sacroiliac joint pain compared to asymptomatic controls (MV - case 13.2, control 8.0, p < 0.05. RR - case 17.4, control 9.5, p < 0.05). No significant difference between those with sacroiliac joint pain and asymptomatic controls in MV and RR (during rest, active straight leg raise with compression), and TV (during rest, active straight leg with and without compression).
Park et al. [95], 2014, Korea, National Research Foundation of Korea, Catholic University of Daegu	Nail salon workers and office worker controls, 264 (256 F, 8 M), 32.9 ± 7.8 y	Inclusion: Nail salon technicians or office workers in Daegu, Korea. Exclusion: NR. Controls (office workers) were matched to cases (nail salon technicians) on NR.	(1) Back Pain, Neck Pain: Point prevalence of MSK symptoms in the neck, upper back, low back assessed with a questionnaire (PRO). (2) IAQ: Exposure to organic chemicals at work in air or handling: yes/no (work classification).	Logistic regression. Exposure to organic chemicals via handling or airborne (nail salon technicians) was associated with a higher risk for MSK symptoms in the neck and upper back compared to non-exposed controls (neck: OR 19.7, 95% CI 8.9-43.6, p < 0.05) (upper back: OR 12.5, 95% CI 3.8-41.0, p < 0.05). No significant relationship between exposure to organic chemicals via handling or airborne and MSK symptoms in the low back.
Park et al. [97], 2020, South Korea, Soonchun-Hyang University Research Fund	Individuals who underwent spine surgery for lumbar degenerative disc disease, 78,241 (41,737 F, 36,504 M), median 55-64 y, NA, NA	Individuals in South Korea who underwent spine surgery for lumbar degenerative disc disease between 2007-2008, claims data available Health Service Review and Assessment Service database. Exclusion: Received reoperation within 1 y of initial surgery.	(1) Back Pain: Reoperation rate following initial spine surgery for lumbar degenerative disc disease assessed via medical chart review: yes/no. (2) Respiratory Disorder: Prevalence of pulmonary disease assessed via medical chart review: yes/no.	Cox proportional hazard regression. The presence of pulmonary disease was significantly associated with an increased risk of reoperation for lumbar degenerative disc disease (HR 1.20, 95% CI 1.15-1.26, p < 0.01).
Park et al. [99], 2023, South Korea, No extramural funding	Residents of South Korea, 17,038 (9,836 F, 7,202 M), 49.2 ± 16.1 y, NA, NA	Inclusion: Residents of South Korea, participants in the Korea National Health and Nutrition Examination Survey, completed chronic low back pain examination survey, age 10-100 y. Exclusion: Missing data on demographic and health questionnaires.	(1) Back Pain: Prevalence of chronic low back pain assessed via questionnaire: yes/no (PRO). (2) Respiratory Disorder: Prevalence of asthma and COPD assessed by questionnaire: yes/no (PRO).	Logistic regression. Asthma and COPD were significantly associated with an increased risk of chronic low back pain over the past 12 mo (asthma: OR 2.34, 95% CI 1.99-2.76, p < 0.001) (COPD: OR 2.70, 95% CI 1.96-3.72, p < 0.001).
Pisinger et al. [100], 2011, Denmark, Danish Medical Research Council, The Danish Centre for Evaluation and Health Technology Assessment, Novo Nordisk, Copenhagen County, Danish Heart Foundation, The Danish Pharmaceutical Association, Augustinus Foundation, Becket Foundation, Henriksens Foundation	General population, 6,784 (3,496 F, 3,288 M), 30-60 y, NA, NA	Inclusion: Adults in Denmark who participated in the Inter99 study. Exclusion: NR.	(1) Back Pain: Back pain and related symptoms in the past 12 mo, a 6-item questionnaire with a 4-point Likert scale, and categorized to a dichotomous variable: yes/no (PRO). (2) IAQ: ETS exposure assessed via questionnaire: How many hours a day do you usually spend in rooms where people smoke?" (almost never, 1/2 - 1 hr; 1 - 5 hr; > 5 hr) (PRO).	Logistic regression. In non-smokers, exposure to ETS ≥ 5 hr/day was significantly associated with an increased risk of low back pain and related symptoms (OR 1.46, 95% CI 1.2-1.8, p < 0.05).
Rasmussen-Barr et al. [101], 2019, Sweden, NR	General population with asthma or COPD from Sweden, 1,301 (747 F, 544 M), 32-40 y (median), NA, NA	Inclusion: General population from Sweden with data available within Stockholm Public Health Cohort, age ≥ 18 y, history of low back pain in past 6 mo ≤ 2 d / mo, history of asthma or COPD. Exclusion: History of low back pain in past 6 mo > 2 d / mo.	(1) Back Pain: Troublesome low back pain in the past 6 mo: yes/no (PRO). (2) Respiratory Disorder: Prevalence of asthma or COPD assessed within health questionnaire: yes/no (PRO).	Binomial regression. Baseline asthma and COPD were significantly associated with an increased risk of troublesome low back pain at 4-y follow-up (asthma: RR 1.29, 95% CI 0.92-1.81) (COPD: RR 2.0, 95% CI 1.13-3.36, p < 0.05).
Rathinaraj et al. [102], 2017, India, No extramural funding	Adults with low back pain, 100 (50 F, 50 M), 23.0 ± 1.6 y, Case (n = 100): Low back pain > 3 mo, Control: NA (comparison to expected value)	Inclusion: Age 20-50 y, low back pain > 3 mo. Exclusion: History of respiratory disorder, cardiothoracic surgery, lung cancer. Controls were matched to cases on: Not applicable (comparison to expected value).	(1) Back Pain: NR. (2) Pulmonary function assessed with spirometer: FEV1.0.	One sample T-test. FEV1.0 was significantly lower in those with chronic low back pain compared to asymptomatic controls (case 2.0 ± 0.3, control 3.3 ± 0.3, p < 0.05).
Ravibabu et al. [103], 2019, India, Indian Council of Medical Research	Battery manufacturing plant workers and office worker controls, 256 (0 F, 256 M), 37.0 ± 7.0 y, Case (n = 176): Lead battery manufacturing plant workers, Exposure to lead at work. Control (n = 80): Office workers, No exposure to lead at work	Inclusion: Workers of lead battery manufacturing plant and exposed to lead with > 2 y experience or office workers. Exclusion: NR. Controls (office workers) were matched to cases on age and socioeconomic status.	(1) Back Pain, Neck Pain: Prevalence of neck, upper back, lower back pain: NMQ (PRO). (2) IAQ: Exposure to lead at work: yes/no (work classification). Quantitative assessment of serum blood lead levels.	Chi-square. Prevalence of MSK symptoms was greater in workers exposed to lead compared to non-exposed controls in the neck, upper back, and low back (neck - exposed: 14%, controls: 4%, p = 0.015) (upper back - exposed: 7%, controls: 0%, p = 0.021) (low back - exposed: 33%, controls: 0%, p = 0.0008). Blood lead levels (µg/dL) were higher in those with pain compared to those without pain in the upper back and low back (upper back - yes: 36.8 ± 16, no: 28.0 ± 13, p < 0.05) (low back - yes: 33.1 ± 13, no: 26.2 ± 13, p < 0.05). No difference in blood lead levels between those with and without pain in the neck.
Roussel et al. [104], 2009, Belgium, Artesis University	Adults with and without low back pain, 20 (10 F, 10 M), 41.4 ± 7.2 y, Case (n = 10): Low back pain > 3 mo, Control (n = 10): No history of low back pain	Inclusion: Age 18-65 y, low back pain > 3 mo of sufficient intensity to limit function and seek treatment. Exclusion: Low back pain related to trauma or pathology, respiratory disease. Controls (without a history of low back pain) were matched to cases on NR.	(1) Back Pain: Clinical assessment, questionnaire (PRO). (2) Pulmonary function: Breathing pattern assessed with pressure biofeedback unit.	Mann-Whitney U test, Fisher exact test. The rate of abnormal breathing patterns during standing deep breathing, active straight leg raise, and bent knee fallout was significantly higher in those with chronic low back pain compared to asymptomatic controls (standing deep breathing - case 60%, control 10%, p = 0.03) (active straight leg raise left - case 50%, control 10%, p = 0.05) (active straight leg raise right - case 50%, control 0%, p = 0.01) (bent knee fall out left - case 60%, case 0% p < 0.01) (bent knee fall out right - case 70%, case 0% p < 0.01). No significant difference between those with chronic low back pain and asymptomatic controls in the rate of breathing patterns at rest in the supine position, standing spontaneous quiet breathing, supine spontaneous quiet breathing, and supine deep breathing.
Shah et al. [105], 2019, India, No extramural funding	Adults with and without low back pain, 28 (14 F, 14 M), 32.2 ± 6.4 y, Case (n = 14): Low back pain > 3 mo, Control (n = 14): No history of low back pain,	Inclusion: Age 18-45 y, low back pain > 3 mo over past 12 mo. Exclusion: Current respiratory complaints, history of low back trauma or surgery in the past 2 mo, cardiovascular disorders, smoking, menstruation. Controls (without a history of low back pain) were matched to cases on age, height, weight, and body surface area.	(1) Back Pain: Pain intensity assessed with NPRS (PRO). (2) Pulmonary function assessed with spirometer: MVV predicted, MVV %predicted, MVV observed, RR; and capnography: PETCO2.	T-test, Wilcoxon's Sign Rank test. MVV predicted and observed were significantly lower in those with chronic low back pain compared to asymptomatic controls (MVV predicted - case 111.0 ± 18.0, control 127.0 ± 13.9, p = 0.015) (MVV observed - case 92.0 ± 27.7, control 124.0 ± 33.8, p = 0.028). No significant difference between those with chronic low back pain and asymptomatic controls in MVV %predicted, PETCO2 sitting, PETCO2 standing, PETCO2 BOSU ball, RR sitting, RR standing, RR BOSU ball.
Smith et al. [106], 2006, Australia, Commonwealth Department of Health and Ageing, National Health and Medical Research Council, NHMRC of Australia	Residents of Australia, 38,050 (38,050 F, 0 M), NR, NA, NA	Inclusion: Residents of Australia, female, participants in the Australian Longitudinal Study on Women’s Health. Exclusion: Pregnant, fracture in previous 12 mo, history of cancer, survey did not provide back pain data.	(1) Back Pain: Prevalence of back pain in the past 12 mo assessed via questionnaire: never/rarely/sometimes/often (PRO). (2) Respiratory Disorder: Prevalence of breathing difficulty in the past 12 mo assessed via questionnaire: never/rarely/sometimes/often (PRO).	Logistic regression. Having breathing difficulty often was significantly associated with an increased risk of having back pain over the past 12 mo in mid-aged and older women (mid-age: OR 2.0, 95% CI 1.4-2.9, p < 0.05) (older: OR 1.9, 95% CI 1.4-2.4, p < 0.05).
Smith et al. [107], 2009, Australia, Commonwealth Department of Health and Ageing, National Health and Medical Research Council, NHMRC of Australia	Residents of Australia, 6,799 (6,799 F, 0 M), 45-50 y (median), NA, NA	Inclusion: Residents of Australia, female, participants in the Australian Longitudinal Study on Women’s Health. Exclusion: Back pain in previous 12 mo, pregnant, fracture in previous 12 mo, history of cancer, the survey did not provide back pain data.	(1) Back Pain: Prevalence of back pain in the past 12 mo assessed via questionnaire: never/rarely/sometimes/often (PRO). (2) Respiratory Disorder: Prevalence of respiratory condition in past 12 mo assessed via questionnaire: absent/present/developed (PRO).	Logistic regression. Breathing difficulty at baseline was significantly associated with an increased risk of developing back pain over 12 mo in mid-age and older women (mid-age: OR 1.63, 95% CI 1.31-2.02, p < 0.05) (older: OR 2.11, 95% CI 1.49-2.99, p < 0.001). Asthma at baseline was significantly associated with an increased risk of developing back pain over 12 mo in younger women (OR 1.31, 95% CI 1.14-1.52, p < 0.001).
Svensson et al. [108], 1983, Sweden, Swedish Work Environment Fund	Residents of Sweden, 940 (0 F, 940 M), 40-47 y, NA, NA	Inclusion: Residents of Goteburg Sweden, name available in city's census register, male. Exclusion: NR.	(1) Back Pain: Prevalence of low back pain assessed via questionnaire: never, at some point in life, ≥ 1 episode per mo, daily episodes, or recurring ≥ 2 episodes per week (PRO). (2) Respiratory Disorder: Prevalence of breathlessness on exertion assessed via questionnaire: yes/no (PRO).	Pitman test. The presence of breathlessness on exertion was significantly associated with an increased risk of low back pain (% breathlessness - never low back pain: 12.7%; low back pain at some point in life: 19.0%; low back pain ≥ 1 episode per mo: 21.3%; low back pain daily episodes or recurring ≥ 2 episodes per week: 24.0%; p < 0.05).
Ulger [109], 2021, Turkey, No extramural funding	Patients undergoing lumbar MRI procedures, 360 (226 F, 134 M), 47.2 ± 12.6 y, NA, NA	Inclusion: Patients undergoing lumbar MRI procedures. Exclusion: History of spinal surgery or instrumentation, spinal fracture or trauma, congenital or acquired spinal deformity tumor or infection, MRI data unavailable, heavy lifting job demands, athletes.	(1) Back Pain: Radiographic assessment via MRI of severe lumbar disc degeneration and classified as a dichotomous variable: yes/no (PRO). (2) IAQ: Exposure to ETS defined as exposure to cigarette smoke for ≥ 1 hr/day for > 1 y assessed via interview: yes/no (PRO).	Logistic regression. In non-smokers, exposure to ETS was significantly associated with increased risk of lumbar disc degeneration at L2-3 and L3-4 levels (L2-3: OR 2.82, 95% CI 1.17-6.80, p = 0.021) (L3-4: OR 3.42, 95% CI 1.53-7.67, p = 0.003). No significant relationship between exposure to ETS and lumbar disc degeneration at L1-2, L4-5, and L5-S1 levels.
Wickstrom et al. [110], 1998, Finland, NR	Shipyard and ventilation factory workers, 306 (0 F, 306 M), 24-55 y, NA, NA	Inclusion: White-collar (n = 117) and blue-collar (n = 189) employees from 2 companies (shipyard and ventilation factories) in Finland. Exclusion: NR	(1) Back Pain: Back pain over the past 12 mo assessed with a questionnaire: yes/no (PRO). Sick leave related to back pain for a number of days. (2) IAQ: Work-related questionnaire with numerous items on the ndoor environment including air draft: 3-point Likert scale (PRO).	Logistic regression. Blue collar workers - At baseline and 24-mo follow-up, air draft was significantly associated with increased risk of back pain over past 12 mo (baseline - OR 2.06, 95% CI 1.11-3.84, p < 0.05) (24-mo - OR 2.00, 95% CI 1.02-3.90, p < 0.05). Sick leave: No significant relationships between air draft and sick leave related to back pain. White collar workers - At baseline and 24-mo follow-up, no significant relationships between air draft with back pain over past 12 mo. Sick leave: NR.
Wirth et al. [111], 2014, Switzerland, NR	Adults with and without neck pain, 38 (28 F, 14 M), 46.5 ± 10.2 y, Case (n = 19): Neck pain > 6 mo, Control (n = 19): No neck pain	Inclusion: Age > 18 y, neck pain > 6 mo assessed by NDI. Exclusion: History of spine fracture or surgery, cervical neurological or inflammatory condition. Controls (without neck pain) were matched to cases of gender.	(1) Neck Pain: Disability assessed with NDI (PRO). (2) Pulmonary function assessed with spirometer: MVV, MEP, MIP, VC, FVC, FEV1.0/FVC, and PEF.	T-test. No significant difference between those with chronic neck pain and asymptomatic controls in MVV, MEP, MIP, VC, FVC, FEV1.0/FVC, PEF.
Wright et al. [112], 1995, United Kingdom, NR	Residents of 19 health districts of North West England, 34,141 (19,097 F, 15,044 M), age 40-64 y (median), NA., NA	Inclusion: Resident of 19 health districts of North West England, completed North West Regional Health Needs Survey, age ≥ 18 y. Exclusion: NR.	(1) Back Pain: Low back pain in the past 12 mo for which doctor was consulted: yes/no (PRO). (2) Respiratory Disorder: Prevalence of respiratory disorders assessed via questionnaire: yes/no (PRO).	Logistic regression. The risk of consulting a doctor for back pain was significantly greater in those with respiratory disorders than those without (OR 1.36, 95% CI 1.19-1.56).
Yalcinkaya et al. [113], 2017, Turkey, Afyon Kocatepe University	Adults with and without neck pain, 160 (80 F, 80 M), 42.7 ± 9.1 y. Case (n = 80): Neck pain ≥ 3 mo, Control (n = 80): No neck pain	Inclusion: Neck pain ≥ 3 mo for ≥ 5d/wk, referred to physical medicine and rehabilitation department for neck pain. Exclusion: Exercise-related risks, cardiopulmonary and renal disease, diabetes, malignancy, infection, dehydration, medications that may affect heart rate, disc herniation with neurological deficits, psychiatric disorders, scoliosis, spondylolisthesis, osteoporosis, rheumatic diseases, treatment in past 3 mo. Controls (without neck pain) were matched to cases on gender and BMI.	(1) Neck Pain: Disability assessed with NDI (PRO). (2) Pulmonary function assessed with spirometer: FVC, FEV1.0, FEV1.0/FVC, PEF, PEF25-75, MVV.	T-test, Mann-Whitney U test. No significant difference between those with chronic neck pain and asymptomatic controls in FVC, FEV1.0, FEV1.0/FVC, PEF, PEF25-75, MVV.
Yeung et al. [114], 2011, China, NR	Personal care workers, 36 (Gender: NR), NR, NA, NA	Inclusion: Personal care workers at one older adult resident facility in China. Exclusion: Disabling low back pain that required sick leave for 7 days prior to testing, history of spinal surgery, metabolic illness, cardiovascular disorders (e.g., rheumatoid arthritis, cardiovascular disease, diabetes, hypertension, or malignancy), severe herniated intervertebral disc.	(1) Back Pain: Pain assessed with NMQ (PRO). (2) IAQ: Work-related questionnaire with numerous items on indoor environment including ventilation: yes/no (PRO).	Logistic regression. No significant relationship between ventilation at work and low back pain.

**Table 5:** Supplemental table: Characteristics and outcomes of included randomized controlled trials General - ANCOVA: Analysis of Covariance; ANOVA: Analysis of Variance; BMI: Body Mass Index; CI: Confidence Interval; COPD: Chronic Obstructive Pulmonary Disease; CT: Computed Tomography; ETS: Environmental Tobacco Smoke; F: Female; Hr: Hour; IAQ: Indoor Air Quality; M: Male; MANOVA: Multivariate Analysis of Variance; Mo: Month; MSD: Musculoskeletal Disorder; MSK: Musculoskeletal; NA: Not Applicable; NDI: Neck Disability Index; NMQ: Nordic Musculoskeletal Questionnaire (original or modified); NPRS: Numerical Pain Rating Scale; NR: Not Reported; NRS: Numerical Rating Scale; NS: Not Significant (p > 0.05); ODI: Oswestry Disability Index (original or modified); OR: Odds Ratio; PR: Prevalence Ratio; PRO: Patient Reported Outcome; RMDQ: Roland Morris Disability Questionnaire; RPE: Rating of Perceived Exertion; RR: Relative Risk; VAS: Visual Analog Scale; Wk: Week; Y: Year. Pulmonary function tests - BHT: Breath Hold Time; ERV: Expiratory Reserve Volume; ERV: Expiratory Reserve Volume; ETCO2: End-Tidal CO2; FEF: Forced Expiratory Flow; FEF25-75: Forced Mid Expiratory Flow; FEV/VC ratio: Ratio of Forced Expiratory Volume to Vital Capacity; FEV/FVC ratio: Ratio of Forced Expiratory Volume to Forced Vital Capacity; FEV: Forced Expiratory Volume; FEV0.5: Forced Expiratory Volume in 0.5 sec; FEV1.0: Forced Expiratory Volume in 1 sec; FEVC: Forced Expiratory Vital Capacity; FIV0.5: Forced Inspiratory Volume in 0.5 sec; FIV1.0: Forced Inspiratory Volume in 1 sec; FIVC: Forced Inspiratory Vital Capacity; FVC: Forced Vital Capacity; IC: Inspiratory Capacity; MEP: Maximum Expiratory Pressure; MIP/MEP ratio: Ratio of Maximum Inspiratory Pressure to Maximum Expiratory Pressure; MIP: Maximum Inspiratory Pressure; MV: Minute Volume; MVV: Maximum Voluntary Ventilation; PEF: Peak Expiratory Flow; PEFR: Peak Expiratory Flow Rate; PETCO2: Partial Pressure of End-Tidal CO2; PFT: Pulmonary Function Test; PIF: Peak Inspiratory Flow; RR: Respiration Rate; TV: Tidal Volume; VC: Vital Capacity.

Author, Year, Country, Funding Source, Population Studied, Sample Size (Gender), Age	Eligibility Criteria	Intervention. Control	Outcomes Assessed: (1) Back Pain, Neck Pain. (2) Pulmonary Function, IAQ.	Analysis. Results
Ahmadnezhad et al. [48], 2020, Iran, NR, Weightlifting and powerlifting athletes, 47 (24 F, 23 M), 21.9 ± 18 y	Inclusion: Weightlifting or powerlifting athlete, age 18 - 25 y, exercise for ≥ 3 y, 3X/wk, 75 min/session, low back pain of approximately 6 months duration with > 3/10 pain intensity, from Hamedan, Iran. Exclusion: Serious spinal pathology, spinal deformity, orthopedic, neurological, rheumatic diseases, or respiratory disease, acute MSK inflammation, history of spinal surgery, currently taking pain-relieving medications, currently receiving physical therapy, pregnant.	Intervention (n = 24): Inspiratory muscle breathing exercise with a handheld device, 30 breaths at 15 breaths/min, 2X/d, 7d/wk, 8 wk. Initial session supervised by a physical therapist. Control (n = 24): Weightlifting and powerlifting training. details: NR. 8 wk. Supervision: NR.	(1) Back Pain: Pain intensity assessed with VAS (PRO). (2) Pulmonary function assessed with spirometer: VC, FEV1, FVC, FEV/VC ratio.	ANCOVA. Following intervention period, significantly greater reduction in pain intensity was observed in the intervention group compared to control (Intervention: pre 5.99 ± 0.27, post 5.26 ± 0.39; Control: pre 5.52 ± 0.33, post 5.72 ± 0.24; p = 0.03). Significantly greater increases in VC, FVC, FEV1.0/VC, and FEV1.0 were observed the intervention group compared to control (VC: Intervention: pre 3.91 ± 0.15, post 4.28 ± 0.27; Control: pre 4.02 ± 0.11, post 4.07 ± 0.14; p < 0.001) (FVC: Intervention: pre 4.18 ± 0.20, post 4.43 ± 0.24; Control: pre 4.22 ± 0.29, post 4.25 ± 0.28; p < 0.001) (FEV1.0/VC: Intervention: pre 76.68 ± 2.14, post 78.76 ± 1.78; Control: pre 76.95 ± 2.55, post 76.96 ± 2.45; p = 0.03) (FEV1.0: Intervention: pre 4.04 ± 0.09, post 4.17 ± 0.41; Control: pre 4.07 ± 0.09, post 4.08 ± 0.06; p < 0.001).
Anwar et al. [50], 2022, Pakistan, No extramural funding, Individuals with neck pain, 68 (28 F, 40 M), 39.6 ± 5.3 y	Inclusion: Neck pain > 3 mo, willingness to be randomized in the study. Exclusion: Dizziness, neck pain related to trauma, disc lesion, spondylosis, or nerve root, smoking, asthma, depression, obesity, and spinal deformity.	Intervention (n = 34): Routine physical therapy (described for control) + diaphragmatic breathing exercises as follows: Semi-Fowler position, 3 sets of 3 minutes diaphragmatic breathing, and 2 minutes of regular breathing. Supervised by a physical therapist. frequency: NR. 8 wk. Control (n = 34): Routine physical therapy + placebo breathing exercises. 1. Routine physical therapy: Infrared radiation, prone isometric neck exercises: 10 min. 2. Placebo breathing exercises: 15 min. frequency: NR. 8 wk. Unsupervised.	(1) Neck Pain: Pain intensity assessed with VAS (PRO). Disability assessed with NDI (PRO). (2) Pulmonary function assessed with spirometer: FEV1.0, FVC, FEV1.0/FVC ratio.	T-test, Mann-Whitney U Test. Following intervention period, significantly greater reductions in pain intensity and disability were observed in the intervention group compared to control (Pain: Intervention: pre 36.24, post 22.93; Control: pre 34.81, post 47.4; p < 0.001) (Disability: Intervention: pre 13.41 ± 1.58, post 6.56 ± 1.93; Control: pre 13.14 ± 1.76, post 8.36 ± 1.87; p = 0.011). Significantly greater increases in FEV1.0, FVC, and FEV1.0/FVC ratio were observed for the intervention group compared to control (FEV1.0: Intervention: pre 33.28, post 40.47; Control: pre 37.60, post 30.81; p = 0.045) (FVC: Intervention: pre 37.82, post 44.68; Control: pre 33.31, post 26.83; p < 0.001) (FEV1.0/FVC: Intervention: pre 64.41 ± 1.86, post 71.97 ± 2.10; Control: pre 64.31 ± 2.32, post 68.08 ± 2.97; p < 0.001).
Gholami-Borujeni et al. [55], 2021, Iran, NR, Athletes with low back pain, 48 (24 F, 24 M), 21.6 ± 2.0 y	Inclusion: Low back pain ≥ 3 mo, moderate-severe (3-10/10) pain, ≥ 3 y of weightlifting and powerlifting experience. Exclusion: Speciﬁc medical diagnosis other than low back pain, history of spinal surgery, balance problems, cardiovascular and pulmonary diseases, FEV/FVC ratio > 80%, lower extremity disorder, malalignments of spine and extremities, use of pain relief medications, other treatments for low back pain, use of androgenic steroids or other doping medications.	Intervention (n = 24): Inspiratory muscle breathing exercise using a handheld device: 30 breaths at 15 breaths/min, 2X/d, 7d/wk, 8 wk. Progression: Increase intensity by 5% every wk. Supervised by a physical therapist. Continued with regular weightlifting and powerlifting exercise: details: NR. Control (n = 24): Continued with regular weightlifting and powerlifting exercise: details: NR.	(1) Back Pain: Pain intensity assessed with VAS (PRO). Disability assessed with the Athletes Disability Index (PRO). (2) None (exclusion criteria: FEV/FVC ratio).	MANOVA. Following intervention period, significantly greater reduction in pain intensity was observed in the intervention group compared to control (Intervention: pre 6.1, post 4.8; Control: pre 5.8, post 5.6; p < 0.05). No significant difference in disability between the intervention and control groups (Intervention: pre 15.0, post 11.6; Control: pre 15.1, post 12.8; p > 0.05).
Dareh-Deh et al. [62], 2022, Iran, No extramural funding, Individuals with neck pain, 48 (gender: NR), 24.7 ± 2.2 y	Inclusion: Neck pain > 3 mo and rated as moderate worst pain, NDI of 28-45%, forward head posture cervical angle < 50 degrees, smartphone use > 4 hr/d. Exclusion: History of neck or back surgery, neurological signs, rheumatoid arthritis, current use of muscle relaxant medications.	Intervention (n = 21): Cervicothoracic exercises (described for control) + balloon breathing exercises as follows: Ball placed between knees, inhale through the nose in 3-4 sec, slowly exhale into the balloon. 4 sets/session, 2 sessions/d, 3d/wk, 8 wk. Supervised by a physical therapist. Control-1 (n = 21): Cervicothoracic exercises: 7 isometric and stretching exercises, 2 sets/session, 1 session/d, 3d/wk, 8 wk. Supervised by a physical therapist. Other groups (n = 21): Educational pamphlet. Cannot be compared to the intervention group since the independent effect of breathing exercises cannot be determined.	(1) Neck Pain: Pain intensity assessed with VAS (PRO). (2) Resting respiratory rate assessed with NR.	ANCOVA. Following intervention period, no significant difference in pain intensity was observed between intervention and control (Intervention: pre 5.1 ± 1.6, post 1.9 ± 0.6; Control: pre 4.0 ± 1.0, post 1.7 ± 0.9; p > 0.05). A significantly greater reduction in respiratory rate was observed in the intervention group compared to the control (Respiratory Rate: Intervention: pre 22.5 ± 2.3, post 18.2 ± 1.7; Control: pre 23.5 ± 3.1, post 21.4 ± 2.2; p = 0.02).
Diaz et al. [66], 2007, Guatemala, NR, Indigenous Mayan-Indian community-dwelling adults, 504 (504 F, 0 M), 27.8 ± 7.2 y	Inclusion: Indigenous Mayan-Indian community-dwelling adults from the San Marcos district of Guatemala, currently using an open wood fire for cooking, a child aged ≤ 4 months, or a pregnant woman. Exclusion: NR.	Intervention (n = 259): Indoor cooking with plancha stove and smoke ventilation system. 18 mo. Control (n = 245): Indoor cooking with a traditional open fire. 18 mo.	(1) Back Pain: Back pain during the past month: yes/no (PRO). (2) IAQ: Indoor cooking method: plancha stove with smoke ventilation system or traditional open fire. CO levels in exhaled breath assessed with a monitoring instrument.	Regression, Whitney U. Following the intervention period, no significant difference in back pain in the past month was observed between the intervention and control groups (Intervention pre 62.3%, post 18.0%, Control pre 62.2%, post 25.3%, p > 0.05). Median CO in exhaled breath was significantly less in the intervention group compared to control at 6-, 12-, and 18-month follow-up (Intervention pre 8.5, post 5.0; Control pre 9.0, post 7.0; p < 0.05).
Ghavipanje et al. [72], 2022, Iran, No extramural funding, Postpartum women with low back pain, 40 (40 F. 0 M), 29.3 ± 3.8 y	Inclusion: Childbirth with full-term infant and vaginal delivery with discharge from hospital within 2 days postpartum, onset of low back pain during pregnancy or within 3 wk following delivery, low back pain ≥ 5 on 0-11 scale, ODI ≥ 20%. Exclusion: Other treatments or analgesic medication use during pregnancy or study enrollment, trauma, skeletal injuries, osteoarthritis, neurological disorders, chronic inflammatory disorders, malignancy, osteoporosis, cauda equina syndrome, insufficient Persian language skills, missed ≥ 3 consecutive study visits.	Intervention (n = 20): Diaphragmatic breathing exercises: Focus on diaphragmatic breathing while performing "dynamic neuromuscular stabilization" core exercises (similar, but not identical to, control): body weight only, various prone, supine, sitting, standing positions - motor control, flexibility, muscular endurance. 45-60 min/session, 3 sessions/wk supervised by a physical therapist, 3 sessions/wk home. weekly progression. 6 wk. Control (n = 20): General core exercises: body weight only, various prone, supine, sitting, and standing positions - motor control, flexibility, muscular endurance. 45-60 min/session. 3 sessions/wk supervised by a physical therapist, 3 sessions/wk home. weekly progression. 6 wk.	(1) Back Pain: Pain intensity assessed with NPRS (PRO). Disability assessed with ODI (PRO). (2) Pulmonary Function: Resting respiratory rate assessed with videography. Breath holds time (inspiratory and expiratory) was assessed with a stopwatch.	ANOVA. Following the intervention period, significantly greater reductions in pain and disability were observed in the intervention group compared to the control (Pain: Intervention: pre 6.4 ± 1.0, post 2.0 ± 0.9; Control: pre 6.6 ± 1.1, post 4.8 ± 1.1; p < 0.001) (Disability: Intervention: pre 37.0 ± 4.3, post 17.5 ± 3.6; Control: pre 36.0 ± 4.7, post 29.8 ± 7.1; p < 0.001). Significantly greater reduction in respiratory rate and greater increases in inspiratory and expiratory breath hold times were observed in the intervention group compared to control (Respiratory Rate: Intervention: pre 20.3 ± 3.4, post 12.5 ± 0.8; Control: pre 19.8 ± 2.4, post 17.2 ± 2.3; p < 0.001) (Inspiratory Breath Hold Time: Intervention: pre 18.1 ± 2.5, post 29.8 ± 2.3; Control: pre 17.9 ± 2.1, post 22.0 ± 1.5; p < 0.001) (Expiratory Breath Hold Time: Intervention: pre 17.8 ± 3.4, post 28.0 ± 2.4; Control: pre 17.6 ± 2.1, post 21.3 ± 3.1; p < 0.001).
Hallman et al. [74], 2011, Sweden, NR, Adults with neck-shoulder pain, 24 (22 F, 2 M), 40.5 y	Inclusion: Age 20-50 y, neck-shoulder pain ≥ 6 mo with persistent symptoms over the past 6 wk, stress-related pain diagnosed by a psychologist. Exclusion: Use of medications that affect ANS function or pain perception within 2 wk of study enrollment, rheumatic disorders, diabetes, MSK trauma, chronic neurological and endocrine disorders, hypertension, coronary artery disease, substance abuse, BMI > 30.	Intervention (n = 12): Resonant breathing exercises: wk 1 and wk 10 - Resonance breathing assessment. wk 2-9 - Resonant breathing exercises with respiratory pacer and visual heart rate variability biofeedback. Each session - four 5-min training periods with 2-min rest between each. 1 session/wk, 10 wk. Supervised by a psychologist. Between supervised sessions - Home-based paced breathing practice, 15 min/d, 5d/wk. Control (n = 12): No intervention. Wk 1 and wk 10 - Resonance breathing assessment. Supervised by a psychologist.	(1) Neck Pain: Pain intensity assessed with NPRS (PRO). Disability assessed with NDI (PRO). (2) None.	ANOVA. Following the intervention period, no significant differences in pain and disability between the intervention and control groups (Pain: Intervention: pre 2.6 ± 1.3, post 1.7 ± 1.4; Control: pre 2.5 ± 1.1, post 2.0 ± 1.7; p > 0.05) (Disability: Intervention: pre 21.3 ± 7.5, post 14.0 ± 10.0; Control: pre 25.6 ± 15.2, post 20.6 ± 14.4; p > 0.05).
Kavya et al. [80], 2020, India, No extramural funding, Adults with low back pain, 36 (gender: NR), 26 ± 9 y	Inclusion: Age 20-50 y, low back pain ≥ 12 wk, pain intensity < 5/10. Exclusion: Respiratory or cardiovascular pathology, psychological disorder, malignancy, fracture, acute or radiating low back pain.	Intervention-1 (n = 12): Motor control exercises (described for control) + Inspiratory muscle breathing exercises using a respirometer. Intervention-2 (n = 12): Motor control exercises (described for control) + Expiratory muscle breathing exercises using balloon and ball. Both intervention groups: 15 breaths/min, 3 sets of 1 min/session, 1 min rest between sets. 7d/wk, 3 wk. Supervised by a physical therapist during wk 1, unsupervised at home wk 2-3. Control (n = 12): Motor control exercises alone: Four floor-based motor control exercises without external load. 10 reps/exercise, 3 sets/session, 7d/wk, 3 wk. Supervised by a physical therapist during wk 1, unsupervised at home wk 2-3.	(1) Back Pain: Pain intensity assessed with VAS (PRO). Disability assessed with ODI (PRO). (2) None.	ANOVA. Following the intervention period, a significantly greater reduction in pain was observed in the intervention-1 group compared to the intervention-2 and control. No significant difference between intervention-2 and control (Intervention-1: pre 3.8, post 0.5; Intervention-2: pre 3.8, post 1.1; Control: pre 3.6, post 1.7; p < 0.001). No significant difference in disability among intervention-1, intervention-2, and control (Intervention-1: pre 14.3, post 5.3; Intervention-2: pre 17.5, post 7.7; Control: pre 20.8, post 10.9; p > 0.05).
Mehling et al. [89], 2005, United States, US DHHS, Mount Zion Health Fund, Adults with low back pain, 36 (18 F, 10 M, 10 NR), 49.2 ± 2.2 y	Inclusion: Age 20-70 y, continuous low back pain 3-24 mo, seeking primary care for low back pain, fluent in the English language. Exclusion: History of spinal surgery, compensation for back pain, current alcohol or drug dependency, cognitive or psychological impairment, and other pain disorders.	Intervention (n = 18): Home exercises (described for control) + Breathing exercises as follows: Skillful touch-mediated breathing therapy based on Middendorf Breath Institute protocol. Meditative, awareness-based program to enable spontaneous, subtle, unmanipulated breathing patterns. 13 sessions, 45-60 min/session, 2 sessions/wk, 6-8 wk. Supervised by a breathing therapist. Control (n = 18): Multi-modal physical therapy program in clinic + home exercises: Strengthening, motor control, flexibility exercises, joint and soft tissue mobilization. functional task performance, education. 13 sessions, 45-60 min/session, 2 sessions/wk, 6-8 wk. Supervised by a physical therapist. Home exercises: Mode: NR. 20-30 min. Frequency: NR. Unsupervised.	(1) Back Pain: Pain bothersomeness assessed with VAS (PRO). Disability assessed with RMDQ (PRO). (2) None.	ANOVA. Following 6-wk intervention period and 6-mo follow-up, no significant differences in pain and disability were observed between the intervention and control groups (Pain: Intervention: pre 5.15, 6-wk 2.44, 6-mo 3.44; Control: pre 4.37, 6-wk 1.94, 6-mo 1.92; p > 0.05) (Disability: Intervention: pre 6.7, 6-wk 1.88, 6-mo 2.98; Control: pre 6.6, 6-wk 3.47, 6-mo 1.42; p > 0.05)
Oh et al. [92], 2020, Korea, NR, Adults with low back pain, 44 (44 F, 0 M), 45.3 ± 2.6 y	Inclusion: Age 40-40 y, female, low back pain in past 6 wk, pain intensity ≥ 3/10 or ≥ 3 positive lumbar instability tests. Exclusion: history of back surgery, pain-related motor performance difficulties, systemic diseases, respiratory diseases, a malignancy, participated in < 85% of study visits.	Intervention (n = 22): Motor control exercises (described for control) + breathing resistance exercise as follows: Breathing with respiratory resistance using a mouth device during motor control exercises. RPE maintained at below 14. 50 min/session, 3X/wk, 4 wk. Supervised by a physical therapist. Control (n = 22): Motor control exercises: 5 motor control exercises on the floor using bodyweight for resistance progressed weekly plus 10 min flexibility exercises. 5 reps/exercise. 3 sets/exercise, 50 min/session, 3X/wk, 4 wk. Supervised by a physical therapist.	(1) Back Pain: Pain intensity assessed with VAS (PRO). Disability assessed with ODI (PRO). (2) Pulmonary function assessed with spirometer: FVC, FEV1.0, FEV1.0/FVC, MVV.	ANOVA. Following the intervention period, a significantly greater reduction in disability was observed in the intervention group compared to the control (Intervention: pre 17.32 ± 5.90, post 9.45 ± 3.92; Control: pre 16.09 ± 4.24, post 10.95 ± 4.36; p = 0.025). No significant difference in pain between the intervention and control groups (Intervention: pre 6.44 ± 0.42, post 4.58 ± 0.46; Control: pre 6.40 ± 0.47, post 4.45 ± 0.42; p > 0.05). Significantly greater increases in FVC, FEV1.0, and MVV were observed in the intervention group compared to control (FVC: Intervention: pre 4.08 ± 0.83, post 4.51 ± 0.83; Control: pre 4.00 ± 0.84, post 4.07 ± 0.83; p < 0.001) (FEV1.0: Intervention: pre 3.07 ± 0.95, post 3.40 ± 0.94; Control: pre 2.84 ± 0.86, post 2.83 ± 0.86; p < 0.001) (MVV: Intervention: pre 87.15 ± 31.25, post 121.69 ± 29.42; Control: pre 89.32 ± 30.53, post 117.62 ± 27.40; p < 0.001). No significant difference in FEV1.0/FVC between the intervention and control groups (FEV1.0/FVC: Intervention: pre 87.50 ± 9.73, post 87.50 ± 9.72; Control: pre 83.09 ± 13.68, post 83.10 ± 13.72; p > 0.05).
Otadi et al. [94], 2021, Iran, Tehran University of Medical Sciences, Athletes with low back pain, 26 (12 F, 12 M, 2 NR), 35.2 ± 9.9 y	Inclusion: Age 20-59 y, low back pain ≥ 12 wk, pain intensity 3-7/10, active recreational athlete 2-4X/wk for ≥ 3 y. Exclusion: History of lumbar surgery, spinal inflammatory disease or deformity, neurological radiating pain, inability to perform study exercises.	Intervention (n = 13): Transcutaneous electrical nerve stimulation and education (described for control) + Diaphragm breathing exercises as follows: Crocodile, supine, seated, and 90/90/90 breathing exercises without and with an elastic band. 2 exercises per wk, progressed weekly among the 4 exercises. 10 min/session, 2 sessions/d, 5 d/wk. Supervised 1X/wk by a physical therapist. Control (n = 13): Transcutaneous electrical nerve stimulation + education: Transcutaneous electrical nerve stimulation: 30 min/session, 3X/wk, 4 wk. Education: details: NR.	(1) Back Pain: Pain intensity assessed with NRS (PRO). Disability (function) assessed with Core Outcome Measures Index (PRO). (2) None.	ANOVA. Following the intervention period, a significantly greater reduction in pain was observed in the intervention group compared to the control (Intervention: pre 5.5 ± 1.1, post 1.7 ± 0.6; Control: pre 4.7 ± 1.3, post 3.1 ± 1.3; p < 0.001). No significant difference in disability between the intervention and control groups (Intervention: pre 4.5 ± 0.7, post 1.9 ± 0.9; Control: pre 4.5 ± 0.9, post 2.6 ± 0.6; p > 0.05).
Park et al. [96], 2019, Korea, National Research Foundation of Korea, Adults with low back pain, 46 (19 F, 24 M, 3 NR), 30.8 ± 5.4 y	Inclusion: Age 18-65 y, low back pain in past 6 wk, pain intensity ≥ 3/10, ≥ 3 positive lumbar instability tests, able to stand on 1 leg for ≥ 30 sec. Exclusion: Compression fracture, systemic disease, malignancy, neurological conditions that preclude study participation, participated in < 80% of study visits.	Intervention (n = 23): Motor control exercises (described for control) + breathing resistance exercise as follows: Breathing with respiratory resistance using a mouth device during motor control exercises. RPE maintained at below 14. 40 min/session, 3X/wk, 4 wk. Supervised by a physical therapist. Control (n = 23): Motor control exercises: 5 motor control exercises on the floor using bodyweight for resistance progressed weekly plus 10 min flexibility exercises. 5 reps/exercise. 3 sets/exercise, 40 min/session, 3X/wk, 4 wk. Supervised by a physical therapist.	(1) Back Pain: Pain intensity assessed with NRS (PRO). Disability assessed with ODI (PRO). (2) Pulmonary function assessed with spirometer: FVC, FEV1.0, FEV1.0/FVC, MVV.	T-test. Following the intervention period, no significant differences in pain and disability were observed between the intervention and control groups (Pain: Intervention: pre 6.85 ± 1.23, post 3.60 ± 1.41; Control: pre 6.91 ± 1.24, post 3.65 ± 1.27; p > 0.05). (Disability: Intervention: pre 16.30 ± 6.68, post 9.85 ± 4.81; Control: pre 16.04 ± 4.15, post 11.08 ± 4.30; p > 0.05). Significantly greater increases in FVC and MVV were observed in the intervention group compared to control (FVC: Intervention: pre 3.87 ± 0.98, post 4.10 ± 1.00; Control: pre 3.75 ± 0.81, post 3.81 ± 0.82; p = 0.004) (MVV: Intervention: pre 99.92 ± 24.75, post 124.14 ± 29.77; Control: pre 102.60 ± 26.86, post 111.24 ± 29.84; p = 0.007). No significant difference in FEV1.0 and FEV1.0/FVC between the intervention and control groups (FEV1.0: Intervention: pre 3.41 ± 0.93, post 3.63 ± 0.97; Control: pre 3.20 ± 0.83, post 3.28 ± 0.77; p > 0.05) (FEV1.0/FVC: Intervention: pre 88.15 ± 8.02, post 88.25 ± 8.71; Control: pre 88.80 ± 7.86, post 85.86 ± 8.98; p > 0.05).
Park et al. [98], 2022, Korea, National Research Foundation of Korea, Individuals with low back pain, 48 (19 F, 23 M, 6 NR), 30.8 ± 5.6 y	Inclusion: Low back pain in past 6 wk, pain intensity ≥ 3/10, ≥ 3 positive lumbar instability tests, able to stand on 1 leg for ≥ 30 sec. Exclusion: History of spinal surgery, respiratory disorders, pregnancy, vestibular disorders that preclude study participation, participated in < 80% of study visits.	Intervention (n = 16): Motor control exercises (described for control) + breathing resistance exercise as follows: Breathing with respiratory resistance using a mouth device during motor control exercises. RPE maintained at 13-14. 60 min/session, 3X/wk, 5 wk. Supervised by a physical therapist. Control (n = 23): Motor control exercises: 5 motor control exercises on the floor using bodyweight for resistance progressed weekly plus 10 min flexibility exercises. 5 reps/exercise. 3 sets/exercise, 40 min/session, 3X/wk, 4 wk. Supervised by a physical therapist.	(1) Back Pain: Pain intensity assessed with VAS (PRO). Disability assessed with ODI (PRO). (2) Pulmonary function assessed with spirometer: FVC, FEV1.0, FEV1.0/FVC, MVV, MIP. MEP.	ANOVA. Following the intervention period, significantly greater reductions in pain and disability were observed in the intervention group compared to the control (Pain: Intervention: pre 6.45 ± 0.44, post 4.66 ± 0.40; Control: pre 6.46 ± 0.46, post 5.96 ± 0.87; p < 0.001). (Disability: Intervention: pre 21.43 ± 1.60, post 10.14 ± 1.17; Control: pre 21.14 ± 1.61, post 13.07 ± 1.44; p < 0.001). Significantly greater increases in FVC, FEV1.0, MIP, and MEP were observed in the intervention group compared to control (FVC: Intervention: pre 4.07 ± 0.88, post 4.41 ± 0.85; Control: pre 4.05 ± 0.90, post 4.10 ± 0.89; p = 0.004) (FEV1.0: Intervention: pre 3.21 ± 1.04, post 4.51 ± 1.01; Control: pre 3.43 ± 1.05, post 3.56 ± 0.99; p = 0.032) (MIP: Intervention: pre 56.94 ± 5.97, post 66.51 ± 3.00; Control: pre 56.46 ± 5.99, post 56.98 ± 5.86; p < 0.001) (MEP: Intervention: pre 50.86 ± 5.62, post 59.52 ± 3.48; Control: pre 50.76 ± 5.65, post 51.61 ± 5.84; p < 0.001). No significant difference in FEV1.0/FVC and MVV between the intervention and control groups (FEV1.0/FVC: Intervention: pre 87.73 ± 10.25, post 86.33 ± 10.38; Control: pre 84.73 ± 10.06, post 88.16 ± 5.91; p > 0.05) (MVV: Intervention: pre 104.34 ± 24.66, post 121.76 ± 22.77; Control: pre 105.03 ± 24.64, post 112.04 ± 23.77; p > 0.05).

**Table 6:** Supplemental table: Type and quality of included observational studies Study quality determined by the NIH quality assessment tool for observational cohort and cross-sectional studies, with 14 items: (1) Was the research question or objective in this paper clearly stated? (2) Was the study population clearly specified and defined? (3) Was the participation rate of eligible persons at least 50%? (4) Were all the subjects selected or recruited from the same or similar populations (including the same time period)? Were inclusion and exclusion criteria for being in the study prespecified and applied uniformly to all participants? (5) Was a sample size justification, power description, or variance and effect estimates provided? (6) For the analyses in this paper, were the exposure(s) of interest measured prior to the outcome(s) being measured? (7) Was the timeframe sufficient so that one could reasonably expect to see an association between exposure and outcome if it existed? (8) For exposures that can vary in amount or level, did the study examine different levels of the exposure as related to the outcome (e.g., categories of exposure, or exposure measured as continuous variable)? (9) Were the exposure measures (independent variables) clearly defined, valid, reliable, and implemented consistently across all study participants? (10) Was the exposure(s) assessed more than once over time? (11) Were the outcome measures (dependent variables) clearly defined, valid, reliable, and implemented consistently across all study participants? (12) Were the outcome assessors blinded to the exposure status of participants? (13) Was the loss to follow-up after baseline 20% or less? (14) Were key potential confounding variables measured and adjusted statistically for their impact on the relationship between exposure(s) and outcome(s)? NA: Not Applicable. N: No. NR: Not Reported. Y: Yes. Overall Quality Rating: 0-4: Poor (high risk of bias) 5-9: Fair (between low and high risk of bias) 10-14: Good (low risk of bias).

Author, Year	Study Type (Evidence Level)	Q1	Q2	Q3	Q4	Q5	Q6	Q7	Q8	Q9	Q10	Q11	Q12	Q13	Q14	Overall Quality Score	Overall Quality Rating
Angst et al. [49], 2017	Prospective cohort (2)	Y	Y	N	Y	N	Y	Y	N	Y	Y	Y	NR	N	Y	9	Fair
Astrand et al. [51], 1987	Cross-sectional (4)	Y	Y	Y	Y	N	N	N	N	N	N	N	NR	NA	Y	5	Fair
Awadallah et al. [52], 2021	Case-control (3)	Y	Y	Y	Y	N	N	N	Y	Y	N	Y	NR	NA	N	7	Fair
Beynon et al. [53], 2021	Prospective cohort (2)	Y	Y	NR	Y	N	Y	Y	Y	Y	Y	Y	NR	N	Y	10	Good
Borisut et al. [54], 2021	Case-control (3)	Y	Y	NR	Y	N	N	N	Y	Y	N	Y	NR	NA	Y	7	Fair
Cagnie [56], 2007	Cross-sectional (4)	Y	Y	Y	Y	N	N	N	Y	Y	N	Y	NR	NA	Y	8	Fair
Carnow et al. [57], 1981	Cross-sectional (4)	Y	N	NR	NR	N	N	N	Y	Y	N	N	NR	NA	N	3	Poor
Cheon et al. [58], 2020	Case-control (3)	Y	Y	NR	Y	N	N	N	Y	Y	N	Y	NR	NA	N	6	Fair
Chuang et al. [59], 2008	Cross-sectional (4)	Y	Y	N	Y	Y	N	N	N	Y	N	Y	NR	NA	N	6	Fair
Clark et al. [60], 2014	Prospective cohort (2)	Y	Y	NR	Y	N	Y	Y	N	Y	Y	Y	NR	N	Y	9	Fair
Dağ et al. [61], 2022	Case-control (3)	Y	Y	NR	Y	Y	N	N	Y	Y	N	Y	Y	NA	Y	9	Fair
de Luca et al. [63], 2017	Cross-sectional (4)	Y	Y	Y	Y	N	N	N	N	Y	N	Y	NR	NA	Y	7	Fair
de Miguel-Díez et al. [64], 2018	Case-control (3)	Y	Y	NR	Y	N	N	N	N	Y	N	Y	NR	NA	Y	6	Fair
Dewey et al. [65], 1989	Prospective cohort (2)	Y	Y	Y	Y	N	Y	Y	Y	Y	Y	Y	NR	Y	N	11	Good
Dimitriadis et al. [67], 2013	Case-control (3)	Y	Y	NR	Y	N	N	N	Y	Y	N	Y	NR	NA	Y	7	Fair
Dimitriadis et al. [68], 2014	Case-control (3)	Y	Y	NR	Y	N	N	N	Y	Y	N	Y	NR	NA	Y	7	Fair
Eriksen [69], 2004	Prospective cohort (2)	Y	Y	Y	Y	N	Y	Y	Y	Y	N	Y	NR	Y	Y	11	Good
Fahad et al. [70], 2020	Case-control (3)	Y	Y	NR	Y	N	N	N	Y	Y	N	Y	NR	NA	N	6	Fair
Fuentes-Alonso et al. [71], 2020	Case-control (3)	Y	Y	NR	Y	N	N	N	N	Y	N	Y	NR	NA	Y	6	Fair
Hagins et al. [73], 2011	Case-control (3)	Y	Y	Y	Y	Y	N	N	Y	Y	N	Y	NR	NA	Y	9	Fair
Hamaoui et al. [75], 2002	Case-control (3)	Y	Y	NR	Y	N	N	N	Y	Y	N	Y	NR	NA	N	6	Fair
Hestbaek et al. [76], 2006	Prospective cohort (2)	Y	Y	Y	Y	N	N	Y	Y	Y	Y	Y	NR	N	Y	10	Good
Hurwitz et al. [77], 1999	Cross-sectional (4)	Y	Y	N	Y	Y	N	N	N	Y	N	Y	NR	NA	Y	7	Fair
Ignatius et al. [78], 1993	Cross-sectional (4)	Y	N	Y	NR	N	N	N	N	Y	N	N	NR	NA	N	3	Poor
Kapreli et al. [79], 2009	Case-control (3)	Y	Y	NR	Y	N	N	N	Y	Y	N	Y	NR	NA	Y	7	Fair
Kupeli et al. [81], 2021	Cross-sectional (4)	Y	Y	NR	Y	N	N	N	N	Y	N	Y	NR	NA	N	5	Fair
Lambrechts et al. [82], 2023	Cross-sectional (4)	Y	Y	N	Y	N	N	N	N	Y	N	Y	NR	NA	Y	6	Fair
Lee et al. [83], 2017	Case-control (3)	Y	Y	Y	Y	Y	N	N	N	Y	N	Y	NR	NA	Y	8	Fair
Li et al. [84], 2017	Cross-sectional (4)	Y	Y	N	Y	N	N	N	N	Y	N	Y	NR	NA	Y	6	Fair
López-de-Uralde-Villanueva et al. [85], 2018	Case-control (3)	Y	Y	Y	Y	Y	N	N	Y	Y	N	Y	NR	NA	N	8	Fair
Lu [86], 2008	Cross-sectional (4)	Y	N	NR	Y	N	N	N	N	N	N	N	NR	NA	NR	2	Poor
Lunardi et al. [87], 2011	Case-control (3)	Y	Y	NR	Y	Y	N	N	N	Y	N	Y	Y	NA	Y	8	Fair
Magnavita et al. [88], 2011	Cross-sectional (4)	Y	Y	Y	Y	N	N	N	Y	Y	N	Y	NR	NA	Y	8	Fair
Mohan et al. [90], 2018	Case-control (3)	Y	Y	NR	Y	Y	N	N	Y	Y	N	Y	NR	NA	N	7	Fair
Noormohammadpour et al. [91], 2017	Cross-sectional (4)	Y	Y	NR	Y	N	N	N	NR	Y	N	Y	NR	NA	Y	6	Fair
O'Sullivan et al. [93], 2002	Case-control (3)	Y	Y	NR	Y	N	N	N	Y	Y	N	Y	NR	NA	N	6	Fair
Park et al. [95], 2014	Case-control (3)	Y	Y	NR	Y	N	N	N	N	Y	N	Y	NR	NA	Y	6	Fair
Park et al. [97], 2020	Retrospective cohort (3)	Y	Y	Y	Y	N	Y	Y	N	Y	Y	Y	NR	NA	Y	10	Good
Park et al. [99], 2023	Cross-sectional (4)	Y	Y	Y	Y	Y	N	N	N	Y	N	Y	NR	NA	Y	8	Fair
Pisinger et al. [100], 2011	Cross-sectional (4)	Y	Y	NR	Y	N	N	N	N	Y	N	Y	NR	NA	Y	6	Fair
Rasmussen-Barr et al. [101], 2019	Prospective cohort (2)	Y	Y	NR	Y	N	Y	Y	N	Y	Y	Y	NR	N	Y	9	Fair
Rathinaraj et al. [102], 2017	Case-control (3)	Y	Y	NR	Y	N	N	N	Y	Y	N	Y	NR	NA	N	6	Fair
Ravibabu et al. [103], 2019	Case-control (3)	Y	Y	NR	N	N	N	N	Y	Y	N	N	Y	NA	Y	6	Fair
Roussel et al. [104], 2009	Case-control (3)	Y	Y	NR	Y	Y	N	N	Y	Y	N	Y	Y	NA	N	8	Fair
Shah et al. [105], 2019	Case-control (3)	Y	Y	NR	Y	N	N	N	Y	Y	N	Y	N	NA	N	6	Fair
Smith et al. [106], 2006	Cross-sectional (4)	Y	Y	N	Y	N	N	N	Y	Y	N	Y	NR	NA	Y	7	Fair
Smith et al. [107], 2009	Prospective cohort (2)	Y	Y	NR	Y	N	Y	Y	Y	Y	Y	Y	NR	N	Y	10	Good
Svensson et al. [108], 1983	Cross-sectional (4)	Y	Y	Y	Y	N	N	N	N	Y	N	Y	NR	NA	Y	7	Fair
Ulger [109], 2021	Cross-sectional (4)	Y	Y	NA	Y	N	N	N	N	Y	N	Y	NR	NA	Y	6	Fair
Wickstrom et al. [110], 1998	Prospective cohort (2)	Y	N	Y	Y	N	Y	Y	Y	Y	Y	Y	NR	NR	Y	10	Good
Wirth et al. et al. [111], 2014	Case-control (3)	Y	Y	NR	Y	N	N	N	Y	Y	N	Y	NR	NA	N	6	Fair
Wright et al. [112], 1995	Cross-sectional (4)	Y	Y	Y	Y	N	N	N	N	Y	N	Y	NR	NA	Y	7	Fair
Yalcinkaya et al. [113], 2017	Case-control (3)	Y	Y	NR	Y	N	N	N	Y	Y	N	Y	NR	NA	N	6	Fair
Yeung et al. [114], 2011	Cross-sectional (4)	Y	Y	NR	Y	N	N	N	Y	Y	N	Y	NR	NA	Y	7	Fair

**Table 7:** Supplemental table: Type and quality of included randomized controlled trials (RCTs) Study quality determined by the NIH quality assessment tool for controlled intervention studies, with 14 items: (1) Was the study described as randomized, a randomized trial, a randomized clinical trial, or an RCT? (2) Was the method of randomization adequate (i.e., use of randomly generated assignment)? (3) Was the treatment allocation concealed (so that assignments could not be predicted)? (4) Were study participants and providers blinded to treatment group assignment? (5) Were the people assessing the outcomes blinded to the participants' group assignments? (6) Were the groups similar at baseline on important characteristics that could affect outcomes (e.g., demographics, risk factors, co-morbid conditions)? (7 Was the overall drop-out rate from the study at endpoint 20% or lower of the number allocated to treatment? (8) Was the differential drop-out rate (between treatment groups) at endpoint 15 percentage points or lower? (9) Was there high adherence to the intervention protocols for each treatment group? (10) Were other interventions avoided or similar in the groups (e.g., similar background treatments)? (11) Were outcomes assessed using valid and reliable measures, implemented consistently across all study participants? (12) Did the authors report that the sample size was sufficiently large to be able to detect a difference in the main outcome between groups with at least 80% power? (13) Were outcomes reported or subgroups analyzed prespecified (i.e., identified before analyses were conducted)? (14) Were all randomized participants analyzed in the group to which they were originally assigned, i.e., did they use an intention-to-treat analysis? NA: Not Applicable. N: No. NR: Not Reported. Y: Yes. Overall Quality Rating: 0-4: Poor (high risk of bias) 5-9: Fair (between low and high risk of bias) 10-14: Good (low risk of bias).

Author, Year	Study Type (Evidence Level)	Q1	Q2	Q3	Q4	Q5	Q6	Q7	Q8	Q9	Q10	Q11	Q12	Q13	Q14	Quality Score	Quality Rating
Ahmadnezhad et al. [48], 2020	RCT (2)	Y	Y	NR	N	Y	Y	Y	Y	NR	Y	Y	Y	Y	N	10	Good
Anwar et al. [50], 2022	RCT (2)	Y	Y	Y	N	Y	Y	Y	Y	NR	NR	Y	Y	Y	Y	9	Fair
Gholami-Borujeni et al. [55], 2021	RCT (2)	Y	Y	Y	N	Y	Y	Y	Y	NR	Y	Y	Y	Y	N	11	Good
Dareh-Deh et al. [62], 2022	RCT (2)	Y	Y	Y	N	Y	Y	Y	Y	NR	NR	Y	Y	Y	N	10	Good
Diaz et al. [66], 2007	RCT (2)	Y	Y	N	N	NR	Y	Y	Y	NR	NR	Y	Y	Y	NR	8	Fair
Ghavipanje et al. [72], 2022	RCT (2)	Y	Y	Y	N	Y	Y	Y	Y	Y	NR	Y	N	Y	Y	11	Good
Hallman et al. [74], 2011	RCT (2)	N	NR	NR	N	Y	Y	Y	Y	NR	NR	Y	N	Y	N	6	Fair
Kavya et al. [80], 2020	RCT (2)	Y	Y	Y	N	Y	Y	Y	Y	NR	NR	Y	N	Y	Y	10	Good
Mehling et al. [89], 2005	RCT (2)	Y	Y	Y	N	NR	Y	N	Y	NR	NR	Y	NR	Y	N	7	Fair
Oh et al. [92], 2020	RCT (2)	Y	Y	Y	N	NR	Y	Y	Y	Y	NR	Y	Y	Y	N	10	Good
Otadi et al. [94], 2021	RCT (2)	Y	NR	NR	N	N	Y	Y	Y	NR	NR	Y	Y	Y	N	7	Fair
Park et al. [96], 2019	RCT (2)	Y	Y	Y	N	NR	Y	Y	N	NR	Y	Y	Y	Y	N	9	Fair
Park et al. [98], 2022	RCT (2)	Y	Y	Y	N	NR	Y	Y	Y	Y	NR	Y	Y	Y	N	10	Good

**Table 8:** Supplemental table: PRISMA 2020 checklist

Section and Topic	Item #	Checklist item	Location where the item is reported
TITLE Impact of Indoor Air Quality and Breathing on Back and Neck Pain: A Systematic Review. Authors: Ezequiel D. Gherscovici and John M. Mayer			Page Number from Manuscript
Title	1	Identify the report as a systematic review.	1
ABSTRACT			
Abstract	2	See the PRISMA 2020 for the Abstracts checklist.	1
INTRODUCTION			
Rationale	3	Describe the rationale for the review in the context of existing knowledge.	1, 2
Objectives	4	Provide an explicit statement of the objective(s) or question(s) the review addresses.	2
METHODS			
Eligibility criteria	5	Specify the inclusion and exclusion criteria for the review and how studies were grouped for the syntheses.	2, 3
Information sources	6	Specify all databases, registers, websites, organizations, reference lists, and other sources searched or consulted to identify studies. Specify the date when each source was last searched or consulted.	2, 3
Search strategy	7	Present the full search strategies for all databases, registers, and websites, including any filters and limits used.	2, Supplementary Table 3
Selection process	8	Specify the methods used to decide whether a study met the inclusion criteria of the review, including how many reviewers screened each record and each report retrieved, whether they worked independently, and if applicable, details of automation tools used in the process.	2, 3
Data collection process	9	Specify the methods used to collect data from reports, including how many reviewers collected data from each report, whether they worked independently, any processes for obtaining or confirming data from study investigators, and if applicable, details of automation tools used in the process.	3
Data items	10a	List and define all outcomes for which data were sought. Specify whether all results that were compatible with each outcome domain in each study were sought (e.g. for all measures, time points, analyses), and if not, the methods used to decide which results to collect.	3
	10b	List and define all other variables for which data were sought (e.g. participant and intervention characteristics, funding sources). Describe any assumptions made about any missing or unclear information.	3
Study risk of bias assessment	11	Specify the methods used to assess the risk of bias in the included studies, including details of the tool(s) used, how many reviewers assessed each study and whether they worked independently, and if applicable, details of automation tools used in the process.	4
Effect measures	12	Specify for each outcome the effect measure(s) (e.g. risk ratio, mean difference) used in the synthesis or presentation of results.	3, 4
Synthesis methods	13a	Describe the processes used to decide which studies were eligible for each synthesis (e.g. tabulating the study intervention characteristics and comparing against the planned groups for each synthesis (item #5)).	3, 4
	13b	Describe any methods required to prepare the data for presentation or synthesis, such as handling of missing summary statistics, or data conversions.	3, 4
	13c	Describe any methods used to tabulate or visually display results of individual studies and syntheses.	3, 4, Tables 1, 2, Supplementary Tables 4, 5
	13d	Describe any methods used to synthesize results and provide a rationale for the choice(s). If meta-analysis was performed, describe the model(s), method(s) to identify the presence and extent of statistical heterogeneity, and software package(s) used.	4
	13e	Describe any methods used to explore possible causes of heterogeneity among study results (e.g. subgroup analysis, meta-regression).	not applicable as noted on page 4
	13f	Describe any sensitivity analyses conducted to assess robustness of the synthesized results.	not applicable as noted on page 4
Reporting bias assessment	14	Describe any methods used to assess risk of bias due to missing results in a synthesis (arising from reporting biases).	not applicable as noted on page 4
Certainty assessment	15	Describe any methods used to assess certainty (or confidence) in the body of evidence for an outcome.	4
RESULTS			
Study selection	16a	Describe the results of the search and selection process, from the number of records identified in the search to the number of studies included in the review, ideally using a flow diagram.	4, 5, Figure 1
	16b	Cite studies that might appear to meet the inclusion criteria, but which were excluded, and explain why they were excluded.	5, 6, Figure 1
Study characteristics	17	Cite each included study and present its characteristics.	5, 6 Supplementary Tables 4, 5
Risk of bias in studies	18	Present assessments of risk of bias for each included study.	6, Supplementary Tables 6, 7
Results of individual studies	19	For all outcomes, present, for each study: (a) summary statistics for each group (where appropriate) and (b) an effect estimate and its precision (e.g. confidence/credible interval), ideally using structured tables or plots.	6, 7, 8 Supplementary Table 4, 5
Results of syntheses	20a	For each synthesis, briefly summarise the characteristics and risk of bias among contributing studies.	4, 5, 6, 7, 8 Tables 1, 2, Supplementary Tables 6, 7
	20b	Present results of all statistical syntheses conducted. If meta-analysis was done, present for each the summary estimate and its precision (e.g. confidence/credible interval) and measures of statistical heterogeneity. If comparing groups, describe the direction of the effect.	not applicable as noted on page 4
	20c	Present results of all investigations of possible causes of heterogeneity among study results.	not applicable as noted on page 4
	20d	Present results of all sensitivity analyses conducted to assess the robustness of the synthesized results.	not applicable as noted on page 4
Reporting biases	21	Present assessments of risk of bias due to missing results (arising from reporting biases) for each synthesis assessed.	not applicable as noted on page 4
Certainty of evidence	22	Present assessments of certainty (or confidence) in the body of evidence for each outcome assessed.	6, 7, 8, Tables 1, 2
DISCUSSION			
Discussion	23a	Provide a general interpretation of the results in the context of other evidence.	8, 9
	23b	Discuss any limitations of the evidence included in the review.	9, 10
	23c	Discuss any limitations of the review processes used.	9, 10
	23d	Discuss implications of the results for practice, policy, and future research.	10
OTHER INFORMATION			
Registration and protocol	24a	Provide registration information for the review, including register name and registration number, or state that the review was not registered.	1, 2
	24b	Indicate where the review protocol can be accessed, or state that a protocol was not prepared.	1, 2 (PROSPERO registration)
	24c	Describe and explain any amendments to information provided at registration or in the protocol.	not applicable
Support	25	Describe sources of financial or non-financial support for the review, and the role of the funders or sponsors in the review.	32
Competing interests	26	Declare any competing interests of review authors.	32, 33
Availability of data, code and other materials	27	Report which of the following are publicly available and where they can be found: template data collection forms; data extracted from included studies; data used for all analyses; analytic code; any other materials used in the review.	33
